# Novel Alligator Cathelicidin As-CATH8 Demonstrates Anti-Infective Activity against Clinically Relevant and Crocodylian Bacterial Pathogens

**DOI:** 10.3390/antibiotics11111603

**Published:** 2022-11-11

**Authors:** Felix L. Santana, Karel Estrada, Morgan A. Alford, Bing C. Wu, Melanie Dostert, Lucas Pedraz, Noushin Akhoundsadegh, Pavneet Kalsi, Evan F. Haney, Suzana K. Straus, Gerardo Corzo, Robert E. W. Hancock

**Affiliations:** 1 Departamento de Medicina Molecular y Bioprocesos, Instituto de Biotecnología, Universidad Nacional Autónoma de México, Cuernavaca 62210, Mexico; 2 Centre for Microbial Diseases and Immunity Research, University of British Columbia, Vancouver, BC V6T 1Z4, Canada; 3 Unidad de Secuenciación Masiva y Bioinformática, Instituto de Biotecnología, Universidad Nacional Autónoma de México, Cuernavaca 62210, Mexico; 4 Department of Chemistry, University of British Columbia, Vancouver, BC V6T 1Z1, Canada

**Keywords:** cathelicidins, antimicrobial peptides, LL-37, biofilms, abscess model, skin model, reptiles

## Abstract

Host defense peptides (HDPs) represent an alternative way to address the emergence of antibiotic resistance. Crocodylians are interesting species for the study of these molecules because of their potent immune system, which confers high resistance to infection. Profile hidden Markov models were used to screen the genomes of four crocodylian species for encoded cathelicidins and eighteen novel sequences were identified. Synthetic cathelicidins showed broad spectrum antimicrobial and antibiofilm activity against several clinically important antibiotic-resistant bacteria. In particular, the As-CATH8 cathelicidin showed potent in vitro activity profiles similar to the last-resort antibiotics vancomycin and polymyxin B. In addition, As-CATH8 demonstrated rapid killing of planktonic and biofilm cells, which correlated with its ability to cause cytoplasmic membrane depolarization and permeabilization as well as binding to DNA. As-CATH8 displayed greater antibiofilm activity than the human cathelicidin LL-37 against methicillin-resistant *Staphylococcus aureus* in a human organoid model of biofilm skin infection. Furthermore, As-CATH8 demonstrated strong antibacterial effects in a murine abscess model of high-density bacterial infections against clinical isolates of *S. aureus* and *Acinetobacter baumannii*, two of the most common bacterial species causing skin infections globally. Overall, this work expands the repertoire of cathelicidin peptides known in crocodylians, including one with considerable therapeutic promise for treating common skin infections.

## 1. Introduction

The rise of antibiotic resistance in bacterial pathogens across all drug classes poses a serious global public health issue. Recent data showed that, in 2019, antimicrobial resistance directly caused 1.27 million deaths and was associated with 4.9 million deaths worldwide [1]. Furthermore, human health issues, such as the severe acute respiratory syndrome coronavirus 2 (SARS-CoV-2) global pandemic, have recently aggravated this problem [2].

Especially concerning are bacteria of the ESKAPE group (*Enterococcus faecium*, *Staphylococcus aureus*, *Klebsiella pneumoniae*, *Acinetobacter baumannii*, *Pseudomonas aeruginosa*, *Enterobacter* sp.) [3]. These recalcitrant pathogens have been classified by the World Health Organization (WHO) as priorities for the development of new treatments, given their high levels of resistance to almost all common antibiotics and their substantial impact on human health worldwide [3,4]. Perhaps underappreciated is the fact that many of these ESKAPE bacteria can form biofilms, bacterial communities that are responsible for over 65% of bacterial infections in the clinics and are more resistant to conventional antibiotic treatments and many host immune responses [5,6]. Consequently, biofilm infections often become persistent and must be dealt with by surgical debridement [6]. Therefore, to adequately address infections due to ESKAPE pathogens, it is essential to identify antimicrobial compounds with activity against both the planktonic and biofilm growth state.

Host defense peptides (HDPs) have been described as an alternative to conventional antibiotics [7,8]. HDPs have broad-spectrum activity against free-swimming (planktonic) bacteria and in some instances biofilms, multitarget mechanisms of action that ensure a lower propensity to induce resistance, and additional assets such as anti-inflammatory properties [9,10]. Cathelicidins are one of the largest families of HDPs in vertebrates and can be recognized by their conserved pre-pro domains despite a broad diversity of mature sequences and structures [7]. They are multifunctional peptides with great potential for the development of new therapeutic agents, and several cathelicidins, or their derivatives, have been evaluated in clinical trials, including the human cathelicidin LL-37 [11].

Crocodylians (crocodiles, alligators, caimans, gavials and false gavials) possess a robust immune system [12,13] that allows them to deal with environmental microorganisms, as well as potential pathogens that are found in their natural microbiota [14,15]. These include several bacterial species that are pathogenic to humans and belong to the ESKAPE group [14,15,16,17]. Thus, crocodylian cathelicidins (crocCATHs) constitute a potential source of natural HDPs that might prove useful as novel anti-infectives.

Six crocCATHs have been previously identified in the Chinese alligator (*Alligator sinensis*) [18] and one was predicted from the American alligator (*Alligator mississippiensis*) [19]. Most of these peptides displayed moderate broad spectrum antimicrobial activity, as well as immunomodulatory properties [18,20,21]. However, the peptide sequences were identified based on either putative functional genome annotation or by BLAST search in combination with expression analysis at transcriptional level. Although these methodologies made it possible to identify novel sequences in these two species, a comprehensive analysis of cathelicidin sequences encoded in several different crocodylian species is lacking. Furthermore, the antibiofilm activities of crocodylian cathelicidins have been poorly characterized, despite the clinical relevance of biofilm infections in humans.

Profile hidden Markov models (HMMs) of multiple sequence alignments are complex statistical models that capture position-specific information about the likelihood of particular residues and the frequency of insertions/deletions in each position of the alignment [22]. They have shown higher accuracy for the detection of remote homologs compared to other methods, such as BLAST [23]. Using a strategy based on profile HMMs, we identified novel crocCATHs in *A. mississippiensis*, *A. sinensis*, *Crocodylus porosus* and *Gavialis gangeticus* and subsequently synthesized four of these. Synthetic crocCATHs demonstrated broad-spectrum in vitro antimicrobial and antibiofilm activities against several bacterial strains, including clinical isolates of ESKAPE pathogens. In addition, we characterized the antibiofilm properties of these crocCATHs in a human skin organoid model and investigated the in vivo anti-infective activity of As-CATH8, the most potent peptide here identified.

## 2. Results

### 2.1. Bioinformatic Screen of Crocodylian Genomes Identified 18 Novel crocCATHs

Analysis of crocodylian genomes using profile hidden Markov models (HMMs) from an alignment of 140 vertebrate (reptiles, birds and mammals) cathelicidins led to the identification of 18 novel cathelicidin sequences (Supplementary Table S1 and Figure S1). Overall, six sequences were identified in *A. mississippiensis*, two in *A. sinensis*, four in *C. porosus* and another six in *G. gangeticus*. Due to the somewhat low quality of the current genome assemblies, particular identified sequences were missing parts of their N-terminal domain (especially the signal peptide region) and were reported as partial (Supplementary Table S2). Nevertheless, they did contain most of the cathelin domain—including the four well-conserved cysteines (Supplementary Figure S1)—as well as a mature peptide region, and were therefore considered new cathelicidins. Furthermore, according to the NCBI BLASTp tool, most of the new peptide sequences (including the signal peptide and cathelin domain regions) shared high identity with complete cathelicidin sequences from crocodylians (>78%) and other reptiles (>46%). Our work also identified five *A. mississippiensis* sequences with 100% identity with crocCATHs labeled as predicted in the NCBI nucleotide and protein databases.

**Figure 1:**
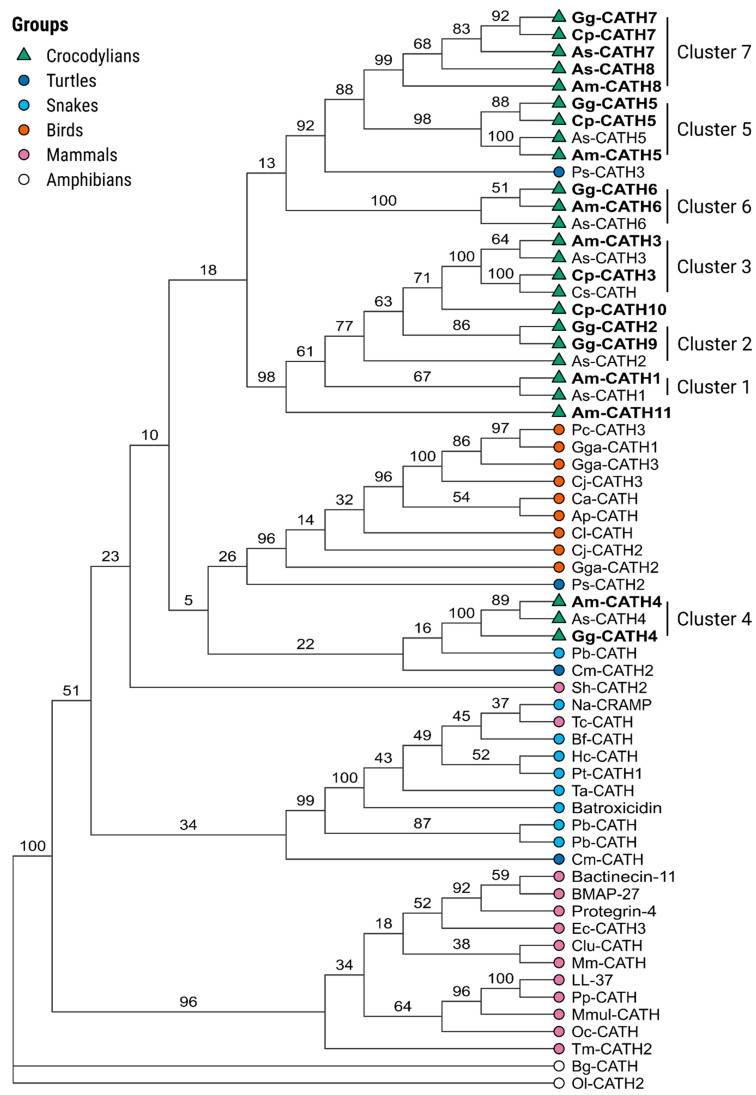
Phylogenetic analysis of crocCATH sequences. The tree was generated using the maximum likelihood criterion implemented in the RaxML program. The analysis included the full-length crocodylian amino acid sequences, as well as sequences from other reptilian, avian and mammalian species. Newly identified crocCATH sequences are displayed in bold. The amphibian cathelicidins Bg-CATH and Ol-CATH2 were used as outgroups to root the tree. Branch numbers indicate statistical support as percent after 1000 bootstrap replicates. The seven identified crocodylian clusters are indicated in the figure, which were named according to the previously described *A. sinensis* cathelicidins [18]. The alligator cathelicidin AM-36 [19] was renamed here as Am-CATH4. NCBI accession numbers of all cathelicidin sequences can be found in Supplementary Tables S1 and S3.

Novel cathelicidins were named according to their orthology with the previously described As-CATH1–6 sequences from *A. sinensis* [18]. Phylogenetic analysis, including sequences from reptiles, birds and mammals, indicated that the crocCATHs grouped into seven well-defined clusters (Figure 1). Interestingly, cluster 4 appeared more distant from most crocCATHs and was grouped together with some snake and turtle sequences, which might indicate that this is the most ancient cluster in the family. Our analysis successfully identified orthologous sequences in most crocodylian clusters. Nevertheless, some cathelicidin sequences were identified with less well-defined orthology relationships (e.g., Am-CATH11 and Cp-CATH10). Moreover, our analysis identified a completely new cluster (cluster 7 in Figure 1) composed of sequences from three crocodylian species, with more distant relationships with Am-CATH8 and As-CATH8. Interestingly, As-CATH7 and As-CATH8 partially overlapped in the same region of the *A. sinensis* genome (Supplementary Table S2).

### 2.2. Mature crocCATHs Displayed Characteristic Properties of α-Helical Cathelicidins

To further study some of the identified crocCATHs, four sequences were selected for chemical synthesis. The sequences As-CATH7 and Gg-CATH7 were selected from the novel cluster 7, whereas the sequence As-CATH8 was selected due to its partial overlap with As-CATH7. The fourth peptide was chosen based on previous studies demonstrating that *A. sinensis* cathelicidin (As-CATH5) has broad-spectrum in vitro antimicrobial activity and is effective against bacterial infections in several in vivo models [18,20,24]. Consequently, the orthologous sequence Gg-CATH5 from *G. gangeticus* was chosen.

The region corresponding to the mature peptide of the selected crocCATHs was manually predicted, as previously described for the As-CATH1–6 sequences [18]. Most of the predicted mature sequences started after a valine residue (Supplementary Table S1), likely part of an elastase cleavage site, located at a residue within positions 138–140 in the multiple sequence alignment (Supplementary Figure S1). The exceptions were the sequences Cp-CATH3 from *C. porosus* and Gg-CATH4 from *G. gangeticus*, which contained an isoleucine and an aspartic acid, respectively. In this case, mature peptides were predicted based on inferred cleavage sites in orthologous cathelicidins from other crocodylian species.

The physicochemical properties of the chosen mature peptides are shown in Table 1. In general, the synthetic crocCATHs were short peptides (20–24 amino acids), with net positive charge (from 3.76 to 4.76) and variable hydrophobicity (from −0.39 to 0.06). Gg-CATH7 displayed the lowest net positive charge, whereas As-CATH8 showed the highest total hydrophobicity index. Notably, all crocodylian peptides displayed somewhat lower net charge and higher hydrophobicity when compared to the human cathelicidin LL-37.

**Figure 2:**
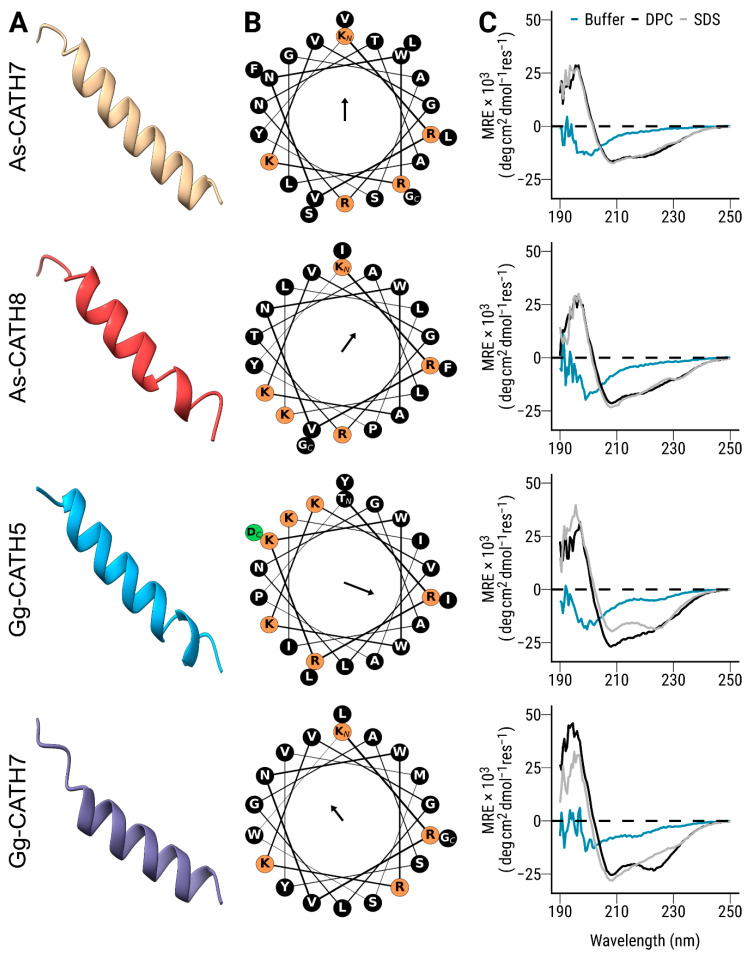
Structural analysis of the synthetic crocCATHs. (**A**) Three-dimensional structures were modeled using the AlphaFold algorithm and visualized in Chimera X. (**B**) Helical wheel representations were generated using the Python package modlAMP. Residues with positive and negative charges are highlighted in orange and green, respectively, whereas the remaining amino acids in the sequence (largely hydrophobic) are shown in black. The orientation of the vector mean hydrophobic moment (amphipathicity) for each sequence is displayed in the center of the wheel. The length of the arrow is proportional to the numerical values shown in Table 1. (**C**) Circular dichroism spectra of crocCATHs were obtained in sodium phosphate buffer (aqua), as well as in the presence of SDS (grey) and DPC (black) micelles. The results are shown as mean residual ellipticity (MRE).

The crocCATHs were structurally characterized using computational tools and circular dichroism spectroscopy. Since the human cathelicidin LL-37 has been extensively characterized as being α-helical [25], it was not included in this analysis. Structural modeling suggested that all four crocCATHs adopted an α-helix conformation (Figure 2A) with an asymmetric distribution of charged and hydrophobic residues on both sides of the helix (Figure 2B), suggesting amphipathicity. Calculation of the mean hydrophobic moments, a numerical estimate of the amphipathicity of a peptide structure [26], indicated differences among the peptides in terms of the magnitude and direction of this vector (Table 1 and Figure 2B). Overall, Gg-CATH5 was the most amphipathic sequence of all crocCATHs, although it showed lower values than those estimated for LL-37 (Table 1).

Circular dichroism spectroscopy studies showed that the synthetic crocCATHs exhibited a largely disordered structure in sodium phosphate buffer (Figure 2C), characterized by low ellipticity above 210 nm and the presence of negative bands near 195 nm in the spectrum [27]. In the presence of dodecyl phosphocholine (DPC, a neutral membrane mimic), all the crocCATHs acquired a helical conformation, as revealed by minima in the ellipticity profile at around 208–210 nm and 222–225 nm (Figure 2C) [27], with some variance from ideal spectra indicating the potential contributions from other structural configurations. Similar spectra were observed in sodium dodecyl sulfate (SDS, an anionic membrane mimic). While cathelicidins can be very diverse in structure [7], crocCATHs displayed similar structural properties to other α-helical members of the cathelicidin family in reptiles, birds and mammals [28,29,30].

### 2.3. As-CATH8 and Gg-CATH5 Exhibited Broad In Vitro Activities against Planktonic and Biofilm Bacteria

The antibacterial activity of the synthetic crocCATHs was evaluated against several medically important pathogens, including those from the ESKAPE group. These peptides showed broad antimicrobial activity against Gram-positive and Gram-negative pathogenic bacteria (Table 2), with the exception of *E. faecium*. In general, minimum inhibitory concentrations (MICs) ranged from 0.25 to 4 μM (~0.6–10 μg/mL). Overall, As-CATH8 and Gg-CATH5 were the most active peptides and showed potency similar to the last-resort antibiotics polymyxin B and vancomycin against Gram-negative and Gram-positive bacteria, respectively, but with activity profiles that overlapped for both types of bacteria. Interestingly, *Proteus vulgaris* was considerably (>8-fold) more susceptible to the cathelicidin peptides As-CATH8 and Gg-CATH5 than to polymyxin B. In addition, these peptides showed better activity than the human cathelicidin LL-37 against *Escherichia coli* and *Salmonella* Typhimurium, with for example, MIC values (in µg/mL) that were 8–16-fold higher than As-CATH8 against both strains (Supplementary Table S4).

The inhibitory activity of the crocCATHs against bacterial biofilms was determined using a microtiter assay against six of these species. The antibiofilm activities showed similar trends to those observed in the MIC assays, although, generally speaking, higher concentrations were required to observe an effect. As-CATH8 was the most active peptide with minimal biofilm inhibitory concentrations (MBIC_95_ in the range of 1–4 μM) against five of six species (except *P. aeruginosa*), while Gg-CATH5 had good activity against four of the six species (Table 3 and Supplementary Figure S2).

The cytotoxicity towards human cells was also investigated and compare to the human cathelicidin LL-37. Lactate dehydrogenase (LDH) release assays showed that As-CATH8 and Gg-CATH5 were moderately cytotoxic against human bronchial epithelial (HBE) cells and peripheral blood mononuclear cells (PBMCs) (Supplementary Figure S3), at the highest concentrations tested (5 and 10 μM). This effect was similar to that observed for the natural human cathelicidin LL-37, which has been used in a phase 1 human clinical trial [31]. In contrast, the crocodylian peptides As-CATH7 and Gg-CATH7 showed low cytotoxicity against both cell types, comparable to the immunomodulatory peptide IDR-1018 used as control.

### 2.4. As-CATH8 and Gg-CATH5 Completely Eradicated S. aureus Biofilms in a Human Organoid Skin Model

Synthetic crocCATHs were tested in a more complex system; namely, a human skin organoid model where bacteria grow as biofilms. In this model, no peptide-induced cytotoxic effects were observed according to LDH release assays and histology studies. In contrast to mono-layer cell models, the skin organoid model demonstrates fully stratified epidermal skin layers, which strongly resemble the morphology and permeability of human skin [32]. Therefore, it is arguably a more relevant model to assess the biological activities of antimicrobial molecules under in vivo-like conditions. Since most crocCATHs had good in vitro activity against *S. aureus*, we tested this organism in the skin model.

**Figure 3:**
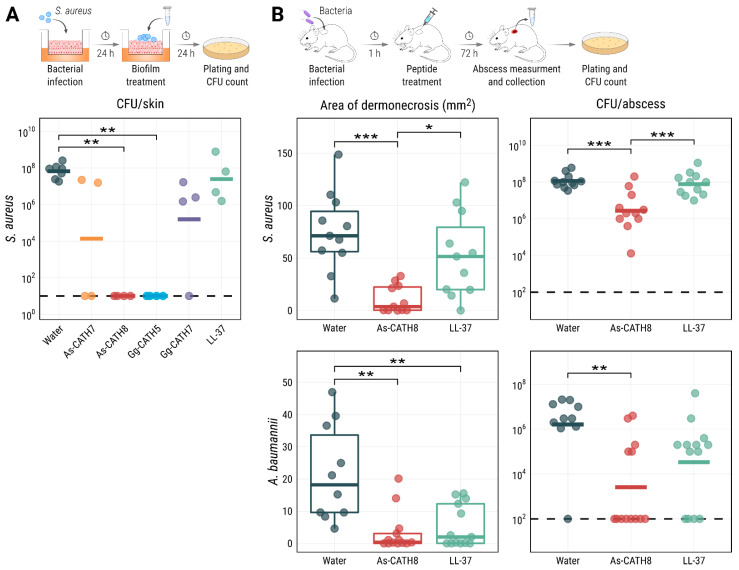
Anti-infective capacity of As-CATH8 and LL-37 in a human skin organoid (**A**) and a murine abscess (**B**) model. (**A**) The ability of crocCATHs to eradicate pre-formed *S. aureus* biofilms was evaluated in a human skin air–liquid interface organoid model. Biofilms were treated with 200 μg of each peptide or with distilled water as a negative control and bacteria were recovered after 24 h. Results are presented as geometric mean per treatment (horizontal bars) of at least four independent biological replicates (dots). Data were statistically analyzed using the Kruskal–Wallis test followed by Dunn’s post hoc test with the Benjamini–Hochberg correction. (**B**) The anti-infective activity of cathelicidin peptides was assessed in a murine abscess model against *S. aureus* and *A. baumannii*. Mice were inoculated with each bacterium for 1 h and then treated intra abscess with 15 mg/kg of peptides or distilled water as a negative control. The area of dermonecrosis and bacterial load in the abscesses was quantified after three days. Results corresponding to the abscess area are shown as box plots and were statistically analyzed using the Kruskal–Wallis test followed by Dunn’s post hoc test with the Benjamini–Hochberg correction. CFU results are shown as geometric mean per treatment (horizontal bars) and were statistically analyzed using ANOVA followed by the Tukey’s post hoc test. Each mouse is represented by individual data points in the abscess experiments. In all the plots, asterisks represent statistically significant differences (* = *p* < 0.05, ** = *p* < 0.01, *** = *p* < 0.001). Detection limits of the bacterial enumeration assays are shown as dashed lines.

The results obtained in the skin-biofilm infection model highlighted the antibiofilm activity of As-CATH8 and Gg-CATH5 cathelicidins (Figure 3A). Treatment with 200 μg of these peptides completely eradicated *S. aureus* biofilms after 24 h. This effect was significant for both peptides when compared to the negative control and was superior to the human cathelicidin LL-37, which showed low and insignificant biofilm eradication activity (3-fold; *p* > 0.05) in this assay. As-CATH7 and Gg-CATH7 had greater antibiofilm activity than LL-37 (4877-fold and 425-fold reductions, respectively) but failed to eliminate bacterial biofilms in most replicates. These results supported As-CATH8 and Gg-CATH5 as the peptides with the highest direct antibiofilm activity of the crocCATHs in this in vivo-like model.

### 2.5. As-CATH8 Showed a Strong Anti-Infective Effect in a Murine Abscess Model

The antibacterial capacities of As-CATH8 and Gg-CATH5 were further evaluated in the murine skin infection/abscess model. Mice were inoculated with clinical isolates of *S. aureus* and *A. baumannii*, two frequent causes of human skin infections [8]. Subcutaneous administration of the crocCATHs or LL-37 alone (15 mg/kg) into uninfected animals showed no evidence of tissue damage or peptide precipitation at the site of injection. Therefore, the peptides were considered non-toxic at the doses used.

In this high-density infection model, Gg-CATH5 showed only weak activity against *S. aureus*. In contrast, As-CATH8 showed a stronger antibacterial effect and was able to substantially decrease the area of dermonecrosis formed above the abscess with both pathogens (Figure 3B). Dermonecrosis is a skin pathology where the skin cells are killed, leaving a visible lesion (the abscess) [33]. The reductions in the *S. aureus* area of dermonecrosis (19-fold) and bacterial burden (42-fold) observed for As-CATH8 were significant when compared to the negative control (distilled water) and LL-37. For the *A. baumannii* infections, treatment with As-CATH8 also reduced both the area of dermonecrosis (61-fold) and the bacterial load (632-fold) when compared to untreated mice (Figure 3B). More importantly, complete eradication of *A. baumannii* was observed in the majority (62%) of mice treated with As-CATH8. Treatment with LL-37 significantly decreased the area of dermonecrosis (ninefold) formed by *A. baumannii* but relatively moderately impacted bacterial recovery in most animals (48-fold).

### 2.6. As-CATH8 Was More Bactericidal and Killed Faster Than Antibiotics

**Figure 4:**
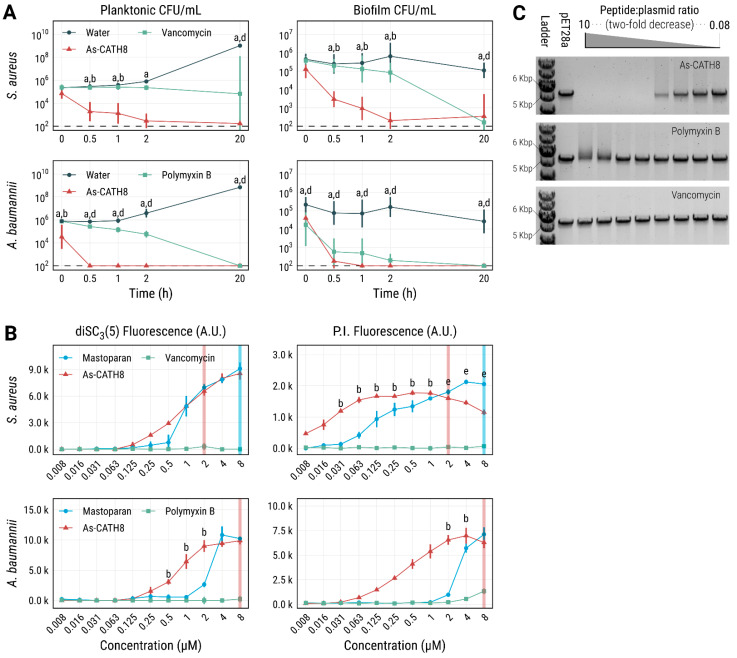
Bacterial killing rate, membrane depolarization and permeabilization and DNA binding capacity of As-CATH8. (**A**) Killing of planktonic *S. aureus* and *A. baumannii* cells at the MIC in MHB media. Antibiofilm activity was assessed in 10% TSB supplemented with 0.1% glucose at 64-fold MIC. CFU data are displayed as geometric mean ×/geometric standard deviation. (**B**) Cytoplasmic membrane depolarization and permeabilization were assessed after 1 h treatment of planktonic cells using DiSC_3_(5) and PI, respectively. Membrane-permeabilizing wasp peptide mastoparan was used as a positive control. Shown are fluorescence readings (mean ± standard error in arbitrary units). Perpendicular lines represent the minimal peptide concentration leading to at least 1000-fold CFU reduction compared to the untreated control (see also Supplementary Figure S4). (**C**) Gel electrophoretic mobility shift assay demonstrating the DNA binding capacity of As-CATH8, employing the linearized plasmid pET28a at 2-fold decreasing peptide:plasmid weight ratios (see Supplementary Figure S5 for an uncropped image). Peptidic antibiotics vancomycin (active against *S. aureus*) and polymyxin B (active against *A. baumannii*) were used as comparisons. Letters denote statistically significant differences (*p* < 0.05) between As-CATH8 and water (a), As-CATH8 and antibiotics (b), As-CATH8 and mastoparan (c), antibiotics and water (d) or antibiotics and Mastoparan (e) according to the Kruskal–Wallis test followed by Dunn’s post hoc test with the Benjamini–Hochberg p-value correction. All experiments were performed at least three times independently.

To further study the antimicrobial activity of As-CATH8, time–kill curves were plotted for planktonic *S. aureus* and *A. baumannii* cells and biofilms (Figure 4A). When compared to vancomycin or polymyxin B, As-CATH8 at its MIC was faster in killing planktonic cells from both bacterial species. Specifically, the crocodylian peptide showed a time-dependent effect on *S. aureus* CFU counts and significantly reduced bacterial survival within 0.5 h of treatment. Furthermore, As-CATH8 was more effective than vancomycin against this bacterium at all time points. While complete killing of planktonic *A. baumannii* by As-CATH8 was seen as soon as 0.5 h after treatment, it took polymyxin B up to 20 h to achieve the same effect.

Biofilm eradication experiments showed similar trends (Figure 4A). In this case, As-CATH8 at 64-fold the MIC nearly eradicated *S. aureus* biofilms within 2 h, while vancomycin showed only a modest effect at that time point. However, vancomycin did show potent activity against *S. aureus* biofilms after 20 h of treatment. As-CATH8 acted rapidly against *A. baumannii* and was able to completely eradicate bacterial biofilms within 1 h of treatment. Notably, polymyxin B showed strong overall activity against this bacterium, although complete eradication was observed only at the 20 h time point.

### 2.7. Potential Role of Interaction with Bacterial Membranes and DNA in the Antibacterial Activity of As-CATH8

To elucidate possible bacterial targets important for the antimicrobial activity of As-CATH8, its effect on bacterial membranes was investigated. Membrane depolarization was assessed using the membrane potential-sensitive dye DiSC_3_(5) (3,3′-dipropylthiadicarbocyanine iodide) [34,35]. This hydrophobic dye with a caged cationic interior can concentrate in bacterial cytoplasmic membranes according to the magnitude of the membrane potential (which is oriented toward the negatively charged interior). A high concentration in the membrane leads to self-quenching of DiSC_3_(5) fluorescence, while depolarization of the bacterial membrane promotes the collapse of the membrane potential, the release of this dye and the subsequent increase of its fluorescence emission [35,36]. In addition, cytoplasmic membrane permeability was investigated using propidium iodide (PI). PI is a commonly used dye that becomes fluorescent when it binds to the DNA. However, it is membrane-impermeant and can only enter cells if cytoplasmic membranes are damaged [36].

The effect of several concentrations of As-CATH8 on *S. aureus* and *A. baumannii* membranes was compared to that of vancomycin and polymyxin B after 1 h treatment (Figure 4B). The membrane-permeabilizing wasp peptide mastoparan [37,38] served as a positive control. While almost no effect was observed for vancomycin and polymyxin B, consistent with a mechanism of action independent of the disruption of the cytoplasmic bacterial membrane [39,40], As-CATH8 showed a concentration-dependent effect on membrane depolarization (DiSC_3_(5) fluorescence) and permeabilization (PI fluorescence) against both bacteria (Figure 4B). This effect was particularly noticeable at concentrations above the MIC of As-CATH8 for *S. aureus* (0.5 μM) and *A. baumannii* (0.25 μM), and it was similar to that observed for mastoparan at high peptide concentrations. As-CATH8 was also able to permeabilize *S. aureus* membranes at concentrations below its MIC (even 16-fold lower). In this case, the PI signal reached the maximal intensity at lower concentrations when compared to *A. baumannii*, for which DiSC_3_(5) and PI signals reached their highest fluorescence values at 2–8 μM As-CATH8 (8- to 32-fold the MIC). Interestingly, maximum values of depolarization or permeabilization did not always lead to notable bacterial killing by As-CATH8, since a 1000-fold reduction in CFU counts was only observed at concentrations higher than 1 μM against *S. aureus* and 8 μM against *A. baumannii* (Figure 4B and Supplementary Figure S4). This suggested that other targets beside the cytoplasmic membrane might be relevant for the mode of action of As-CATH8.

Therefore, the DNA binding capacity of As-CATH8 was evaluated in a gel electrophoretic mobility shift assay [41] using the linearized plasmid pET28a (Figure 4C). The peptidic antibiotics vancomycin and polymyxin B showed no and weak capacities for binding to DNA, respectively. In contrast, As-CATH8 was able to affect DNA migration at peptide:plasmid ratios exceeding 0.63:1. This suggested that, when As-CATH8 enters the cells, it could interact with nucleic acids to affect cellular processes, such as replication, transcription, and translation.

## 3. Discussion

Skin and soft tissue infections (SSTIs) are among the most prevalent bacterial diseases in humans and constitute one of the main precursors of severe sepsis [8]. Furthermore, they pose a significant financial burden on the healthcare system [6]. *S. aureus* has been identified among the most frequent bacteria isolated from SSTIs worldwide, whereas Gram-negative ESKAPE bacteria, such as *A. baumnannii*, are more frequently associated with chronic or postoperative wounds [8]. Treatment of biofilm-associated SSTIs, which usually exhibit higher antibiotic resistance, is particularly difficult [5]. More efficacious treatments are therefore desperately needed. In this context, HDPs have shown promising results for the treatment of this type of infections [8].

Bioinformatic strategies have enabled new possibilities for the identification of HDPs in lesser-studied species, such as crocodylians [42]. Previously, HMMs have been successfully used to explore cathelicidin peptides in several vertebrate species [43,44]. Using a similar strategy, 18 cathelicidins were identified in four crocodylian species (i.e., *A. mississippiensis*, *A. sinensis*, *C. porosus* and *G. gangeticus*), which showed broad and potent antimicrobial activity against most ESKAPE pathogens and *P. vulgaris*. In particular, As-CATH8, the most active crocodylian peptide, showed similar MIC values to the last-resort antibiotic vancomycin and polymyxin B (Table 2). It also impaired biofilm formation in all bacterial strains tested (Table 3). Additionally, As-CATH8 was generally faster than both antibiotics at killing *S. aureus* and *A. baumannii* planktonic cells and pre-formed biofilms (Figure 4A), highlighting the advantages of As-CATH8 compared to vancomycin and polymyxin B in treating infections and limiting the development of bacterial resistance.

It is well-known that HDPs can interact with multiple extracellular and intracellular targets to exert their antimicrobial and antibiofilm effects [9,11]. Experiments using fluorescent probes showed that As-CATH8 was able to depolarize and permeabilize *S. aureus* and *A. baumannii* membranes (Figure 4B). Although it seems likely that compromising the integrity of the membrane is a key component of the killing mechanism of As-CATH8, this may not always be a fatal event and additional or alternative activities might also play a significant role. Two main elements support this idea: first, maximum values of depolarization (DiSC_3_(5) fluorescence) and permeabilization (PI fluorescence) did not always lead to a notable reduction in CFU counts (Supplementary Figure S4), suggesting that bacteria can recover to some extent from this perturbation, as discussed previously for cationic peptides [45]. Second, at concentrations near the MIC, As-CATH8 showed only modest effects on the membranes, while peptide internalization and intracellular signaling may still have occurred at these concentrations. Interaction with bacterial DNA has been shown to be important for the antimicrobial mechanism of several vertebrate cathelicidins, including LL-37 [46,47,48]. This interaction can potentially interfere with several bacterial processes, such as replication and transcription, and usually leads to bacterial death [47,49]. In this study, As-CATH8 displayed stronger DNA binding capacity than vancomycin or polymyxin B, even at peptide ratios equivalent to the MIC against most pathogens (0.8–6.3 μg/mL). Differences in DNA binding affinity between As-CATH8 and antibiotics cannot only be attributed to differences in net charge, since polymyxin B (+5) [50] and As-CATH8 (+4.76) have similar positive charges. In contrast, vancomycin was the least charged of all the molecules (net charge +1) [51] and this could explain the lack of affinity observed for vancomycin to the negatively charged pET28a plasmid (Figure 4C). Taken together, we propose that As-CATH8 kills pathogens through both membrane disruption and alternative mechanisms of action, possibly through interaction with DNA, as suggested for other reptilian cathelicidins [47].

In line with observations for LL-37, cytotoxicity experiments showed that As-CATH8 and Gg-CATH5 were moderately toxic towards HBE cells and PBMCs, whereas As-CATH7 and Gg-CATH7 showed negligible effects. In this regard, other crocodylian cathelicidins have also demonstrated relatively high in vitro cytotoxicity against some cell lines, albeit at higher concentrations than those used here [18]. Although further investigation of their cytotoxic properties is warranted before crocCATHs can be used systemically to treat bacterial infections, it is important to note that in more complex systems, such as the human skin organoid and murine abscess models, no appreciable cytotoxicity was observed upon treatment with As-CATH8 or Gg-CATH5. These results therefore reaffirmed the known limitations of cell lines for assessing peptide cytotoxicity [52] and emphasize the importance of physiologically relevant environments for examining the biological activity of HDPs.

The in vivo anti-infective capacity of As-CATH8 was evaluated in a high-density murine bacterial abscess model. This high-density infection model is difficult to treat with conventional antibiotics but peptides have shown some success. Under these conditions, As-CATH8 decreased the area of dermonecrosis and bacterial load (Figure 3B) in abscesses formed by both *S. aureus* and *A. baumannii* and overall was more effective than the human cathelicidin LL-37. In particular, *A. baumannii* was quite sensitive to treatment with As-CATH8, which caused a reduction of at least 4-fold in bacterial load in most mice (Figure 3B). These results aligned with previous studies that have shown the therapeutic potential of crocCATHs against bacterial infections. For example, the cathelicidin peptides As-CATH2, As-CATH3 and As-CATH5 from *A. sinensis* demonstrated anti-infective properties against *E. coli* and *S. aureus* in a murine peritonitis model [18], while the protective capacity of As-CATH4 and As-CATH5 against bacterial infections was also revealed using invertebrate models [21]. Interestingly, Gg-CATH5, the *G. gangeticus* novel ortholog of the As-CATH5 peptide from *A. sinensis* [18], did not show significant anti-infective capacity as As-CATH8 in the abscess model against *S. aureus*, despite showing similar activity in other assays, including the skin organoid model. Studies involving reptile cathelicidins have suggested that the immunomodulatory activity of these molecules plays an important role in their activity in vivo [18,53,54]. Therefore, the evaluation of As-CATH8 in other animal models that allow the characterization of its immunomodulatory effects at non-toxic concentrations could provide more information about its optimal biological targets.

Overall, this work expands the repertoire of cathelicidins known in crocodylians and highlights the potential of bioinformatic tools to screen reptilian species that are attractive for the identification of natural HDPs. Moreover, we identified the As-CATH8 peptide, which has substantial therapeutic promise for treatment of *S. aureus* and *A. baumannii* skin infections, two of the most prevalent bacterial species causing SSTIs worldwide.

## 4. Material and Methods

### 4.1. Identification of Cathelicidin Sequences Using HMMs

To identify novel crocCATHs, the versions ASM28112v4 (assembly accession number: GCA_000281125.4) for *A. mississippiensis*, ASM45574v1 (assembly accession number: GCA_000455745.1) for *A. sinensis* [55], CroPor_comp1 (assembly accession number: GCA_001723895.1) for *C. porosus* and GavGan_comp1 (assembly accession number: GCA_001723915.1) for *G. gangeticus* were downloaded from NCBI. The initial versions of the genome assemblies generated by St John et al. [56] were also employed.

An automated workflow was established using Snakemake version 5.3.0 [57]. First, profile HMMs were generated using HMMER3 version 3.21 (http://hmmer.org/, accessed on 20 July 2018) from a multiple sequence alignment comprising 140 cathelicidin amino acid sequences of different vertebrate species (reptiles, birds and mammals), retrieved from publicly available sequence databases.

Multiple sequence alignment was performed using MAFFT version 7.310 [58] employing the iterative method with refinement (L-INS-i) and manually curated using the program AliView version 1.26 [59]. Six open reading frames for each version of the genomes were searched for the generated profile HMMs. Matched regions were extracted and expanded 5 kbp upstream and downstream. Expanded regions were then aligned to the initial amino acid sequences using the Exonerate software version 2.4 [60] to obtain the best exon/intron prediction for each crocodylian sequence. These steps were repeated until no new sequences were found, each time starting from a new alignment comprising all crocodylian sequences identified in the previous iterations.

The signal peptide domains in the identified crocCATH sequences were predicted by the SignalP 5.0 server [61]. The prediction of the mature peptide regions was based on information from previously published reptilian cathelicidins [18,62]. Neutrophil elastase was assumed to be the enzyme responsible for processing the mature crocodylian peptides, as suggested for other vertebrate cathelicidins [63,64,65].

### 4.2. Phylogenetic Analysis of the Cathelicidin Sequences

Phylogenetic analysis of the identified crocCATH sequences was performed with RaxML (Randomized Axelerated Maximum Likelihood) software version 8.2.12 [66] using the maximum likelihood criterion. Full-length cathelicidin sequences from various reptilian, avian and mammalian species were also included. The amphibian Ol-CATH2 sequences from *Odorrana livida* (NCBI accession number: AXR75914) and Bg-CATH from *Bufo gargarizans* (NCBI accession number: ANV28414) were used as outgroups to root the tree. The VT + G4 model was adopted as the best amino acid substitution model in accordance with the Aikaike’s and Bayesian information criteria implemented in ModelTest-NG software version 0.1.6 [67]. Rapid analysis with 1000 bootstrap replicates and determination of the highest-scoring maximum likelihood tree was performed in the same run in RaxML. The resulting phylogenetic tree was edited using treeio version 1.18.1 [68] and visualized with ggtree version 3.2.1 [69] in R version 4.2.1 [70] and RStudio version 2022.2.0.443 [71].

### 4.3. Prediction of Physicochemical and Structural Characteristics of Mature crocCATHs

Physicochemical properties of mature cathelicidins including length, net charge and hydrophobicity index were predicted with the Peptides package version 2.4.4 [72] in RStudio. Net charge was estimated using the Bjellqvist scale [73], assuming an environmental pH = 7.0. This pKa scale is based on the polypeptide migration in an immobilized pH gradient. In addition, the hydrophobicity index was calculated using the Kyte–Doolittle scale, which is based on an amalgam of experimental observations derived from the literature [74]. Both the Bjellqvist and the Kyte–Doolittle scales are implemented in extensively used bioinformatic software, such as the ProtParam tool from the ExPASy server [75]. The mean hydrophobic moment was estimated with the modlAMP package version 4.3.0 [76] in Python version 3.9.6, employing an angle of 100°, which is recommended for α-helical structures [26]. Wheel representations of the distribution of hydrophobic and charged residues of the crocCATHs were also generated using the same package.

Three-dimensional models of the crocCATH mature peptides were obtained using a freely available and slightly simplified version of the AlphaFold algorithm version 2.0 [77], which was implemented in Google colab [78]. The structures were visualized in Chimera X version 1.2.5 [79].

### 4.4. Peptides, Reagents and Culture Media

The amino acid sequences of the synthetic crocCATH peptides and the human cathelicidin LL-37 are shown in Table 1. Crocodylian peptides were chemically synthesized by Genscript (Piscataway, NJ, USA), whereas the LL-37 and the innate defense regulator peptide 1018 (VRLIVAVRIWRR-NH_2_) [80] were purchased from CPC Scientific Inc. (Sunnyvale, CA, USA). The wasp venom-derived peptide mastoparan (INLKALAALAKKIL-NH_2_, also named mastoparan-L) [37,81] was acquired from Peptide 2.0 Inc. (Chantilly, VA, USA) All synthetic peptides were obtained with purity higher than 95%.

Peptide stocks were prepared in sterile endotoxin-free water (Baxter, Classic Health, Edmonton, AB, Canada) and adjusted to the desired concentration (usually 2 or 2.5 mM), which was estimated by absorbance at 280 nm in a Nanodrop ND-1000 (ThermoFisher Scientific, Waltham, MA, USA). Concentration values were corrected using the theoretical extinction coefficients of each peptide estimated with the ProtParam tool (https://web.expasy.org/protparam/, accessed on 30 July 2019) from the ExPASy server. Since LL-37 and mastoparan do not contain aromatic residues that absorb at 280 nm, their concentrations were estimated by weight, assuming the lyophilizates contained 70% peptide mass.

The antibiotics polymyxin B and vancomycin were obtained from MilliporeSigma (Burlington, MA, USA) and were resuspended in water to the desired stock concentrations. The DiSC_3_(5) dye was obtained from MilliporeSigma (Burlington, MA, USA) and the PI dye was from ThermoFisher Scientific (Waltham, MA, USA). Working aliquots of DiSC_3_(5) were prepared in 100% DMSO, kept at −20 °C and freeze-thawed no more than three times.

The bacterial culture media Luria-Bertani (LB) broth, brain heart infusion (BHI), tryptic soy broth (TSB), Todd Hewitt broth (THB) and Mueller Hinton broth (MHB) were obtained from ThermoFisher Scientific (Waltham, MA, USA).

All other culture media and supplements used in the human skin organoid model were purchased from MilliporeSigma (Burlington, MA, USA).

### 4.5. Determination of the Secondary Structure of Synthetic Cathelicidins

Circular dichroism experiments were carried out using a JASCO J-815 spectropolarimeter (Jasco, Easton, MD, USA) at room temperature as previously described [82]. All samples were prepared in 25 mM sodium phosphate buffer (pH 7.4) at a final peptide concentration of 100 μM. Spectra were obtained in buffer solution and in the presence of 10 mM sodium dodecyl sulfate (SDS) and 7.5 mM dodecyl phosphocholine (DPC) micelles.

Spectra were corrected by subtracting the buffer background and data were analyzed as mean residual ellipticity values and plotted in RStudio. Final spectra represent an average of three scans.

### 4.6. Bacterial Strains and Culture Conditions

The following bacterial pathogens were used in this study: *S. enterica* subsp. *enterica* serovar Typhimurium ATCC 14028, *E. coli* O157:H7 [83], the clinical isolates *S. aureus* SAP0017 [84] and USA300 LAC [85], *E. faecium* #1-1 [85], *A. baumannii* Ab5075 [85] and *E. cloacae* 218R1 [85], as well as *P. aeruginosa* PAO1 [34], *P. vulgaris* HSC7200-T2 (Hancock Lab strain collection) and *K. pneumoniae* KPLN49 [85].

Overnight cultures were grown in LB at 37 °C with shaking at 250 rpm, except for *E. faecium*, which was grown in BHI medium.

### 4.7. Antimicrobial Activity and Biofilm Inhibition Assays

The antimicrobial activity of the crocCATHs was evaluated with the broth microdilution method as described previously [86]. MIC was defined as the first peptide or antibiotic concentration without visible bacterial growth. Reported values are the statistical mode of at least three independent experiments.

Bacterial biofilm inhibition assays were performed using a microtiter assay as described by Haney et al. [87]. For *E. cloacae*, *E. coli*, *P. aeruginosa* and *S*. Typhimurium, assays were performed using BM2 minimal medium (62 mM potassium phosphate, 7 mM ammonium sulfate, 0.5 mM magnesium sulfate, pH 7.0) supplemented with 0.4% glucose (*w*/*v*). Antibiofilm activity against *A. baumannii* and *S. aureus* SAP0017 was evaluated in 10% TSB (*v*/*v*) media supplemented with 0.1% glucose (*w*/*v*), whereas 10% THB (*v*/*v*) medium was employed for *P. vulgaris*.

Data from at least three independent experiments were analyzed as the percentage of biofilm mass compared to the untreated control. After crystal violet staining, MBIC_95_ was then calculated for each peptide, defined as the minimal peptide concentration capable of inhibiting mean biofilm growth by at least 95% compared to the untreated control [87].

### 4.8. Cell Lines and Peripheral Blood Mononuclear Cells (PBMCs)

Immortalized human bronchial epithelial (HBE) cells (16HBE14o-) were used for in vitro cytotoxicity experiments as described elsewhere [52,88].

The human N/TERT keratinocyte cells used in the skin model experiments were provided by Dr. Peter Nibbering (Leiden University Medical Center, The Netherlands) and cultured as detailed by Wu et al. [32].

Human PBMCs were isolated from human blood following the ethics protocols of the University of British Columbia, Canada. Consent was obtained from healthy volunteers before blood donation. Isolation and treatment of unstimulated PBMC was performed as previously described [80].

### 4.9. Lactate Dehydrogenase (LDH) Release Assays

Peptide-induced cytotoxicity was assessed by measuring the extracellular activity of the LDH enzyme using the Cytotoxicity Detection Kit (MilliporeSigma, Burlington, MA, USA) as previously described [80,88]. The percentage of LDH release relative to the untreated (negative) and Triton X-100 (positive, 100% cytotoxicity) control was assessed for at least three biological replicates and recorded as percent cytotoxicity.

### 4.10. Skin Model Experiments

The human N/TERT epidermal skin models were established as previously published [32]. Briefly, models were cultured in 12-well plates seeded with N/TERT keratinocyte cells at a density of 3 × 10^5^ cells/insert in DermaLife K Keratinocyte complete medium supplemented with LifeFactors (Lifeline Cell Technology, Oceanside, CA, USA). Skin models were cultured at the air–liquid interface for 10 days at 37 °C with 7.3% CO_2_ before being used in experiments.

To investigate the antibiofilm capacity of the cathelicidin peptides, 5 μL (~10^6^ CFU) of *S. aureus* SAP0017 grown to mid-log phase was applied to the center of the N/TERT skin and plates were incubated for 24 h. Skin biofilms were then treated with 30 μL (200 μg) of each crocodylian peptide or the human LL-37 as a comparison. Sterile distilled water was used as a negative control. After 24 h of treatment, skin samples were excised from the culture inserts, sonicated for 5 min in sterile PBS and vortexed for 30 s. Bacterial counts were determined by serial dilution and plating on LB agar. The detection limit of the assay was 10 CFU/skin.

### 4.11. Bacterial Abscess Formation and Peptide Treatment

The animals used in this study were female CD-1 mice purchased from Charles River Laboratories Inc. (Wilmington, MA, USA), 5–7 weeks old and weighing 25 ± 5 g at the time of the experiment. Mice were housed in cohorts of 4–5 littermates exposed to the same pathogen. Standard animal husbandry protocols were employed.

The in vivo activity of As-CATH8 was examined in a subcutaneous abscess infection model as previously described [33] using *S. aureus* USA300 LAC and *A. baumannii*. Fifty microliters of the bacterial culture were injected subcutaneously into the shaved left dorsum of mice at an inoculum density of 5–15 × 10^7^ CFU. One hour later, abscesses were treated with either 15 mg/kg As-CATH8 or LL-37 or sterile endotoxin-free water (Baxter, Classic Health, Edmonton, AB, Canada) as a negative control. Daily clinical grading of the animals was recorded post-treatment for 72 h; mice were then euthanized by exposure to CO_2_ followed by cervical dislocation. Visible dermonecrosis was measured using a caliper and abscesses were harvested in PBS and homogenized using a Mini-Beadbeater (BioSpec Products, Bartlesville, OK, USA). Bacterial counts were determined by serial dilution and plating on LB agar. Two or three independent experiments were performed, each containing 2–4 biological replicates. The detection limit of this model was 100 CFU/abscess.

### 4.12. Planktonic and Biofilm-Killing Assays

The bactericidal activity of As-CATH8 and antibiotics was evaluated against planktonic *S. aureus* SAP0017 and *A. baumannii* cells under similar conditions to those used in the antimicrobial assays. Bacteria were grown overnight in LB media and diluted to ~5 × 10^5^ CFU/mL in MHB. Bacterial cultures (1 mL) were treated with As-CATH8 or antibiotics (vancomycin for *S. aureus* or polymyxin B for *A. baumannii*) at the MIC and tubes were incubated at 37 °C with gentle shaking. Samples were taken at 0, 0.5, 1, 2 and 20 h after treatment and CFU counts determined by serial dilution and plating on LB agar.

Biofilm-killing assays were set up like the biofilm inhibition assays previously described. After forming the biofilms for 24 h in 10% TSB (*v*/*v*) supplemented with 0.1% glucose (*w/v*), *S. aureus* SAP0017 and *A. baumannii* biofilms were treated with As-CATH8 or antibiotics (vancomycin or polymyxin B) at 64 × MIC. Plates were incubated at 37 °C and bacterial biomass was scraped out of the wells with cotton swabs at 0, 0.5, 1, 2 and 20 h after treatment. Cotton tips were placed in 1 mL of LB and sonicated for 5 min, and bacterial numbers were determined by serial dilution and plating on LB agar.

### 4.13. Membrane Depolarization and Permeabilization Assays

The cytoplasmic membrane depolarization and permeabilization activities of As-CATH8 compared to those of antibiotics (vancomycin or polymyxin B) were assessed against *S. aureus* SAP0017 and *A. baumannii* using the membrane-potential sensitive dye DiSC_3_(5) and the cell viability dye PI [36]. The membrane-permeabilizing peptide mastoparan [37] was included as a positive control and water served as a negative (untreated) control.

An end-point assay was performed as suggested by Boix-Lemonche et al. [89] with several modifications. Briefly, after growth in LB, mid-log phase bacteria were centrifuged and washed twice with 5 mM HEPES buffer supplemented with 20 mM glucose (HEPES-Gluc). Cell density was adjusted to ~10^7^ CFU/mL in (HEPES-Gluc) supplemented with 0.1 M KCl. A fraction (10 mL) of the culture was treated with 0.8 μM DiSC_3_(5) and incubated for 1 h at 23 °C protected from the light. Another 10 mL were then treated with 3.5 μM PI and incubated under the same conditions for 15 min. After incubation, 190 μL/well of the cultures were added to black opaque 96-well plates (Corning Inc.) and pre-treatment fluorescence was monitored every 2 min for a total of 10 min using a Synergy H1 Hybrid Multi-Mode Reader (BioTek, Winooski, VT, USA). The excitation and emission wavelengths were the following: for DiSC_3_(5), 305 nm excitation and 617 nm emission; for PI, 622 nm excitation and 700 nm emission. The digital gain was adjusted to 150/255 for DiSC3(5) and 110/255 for PI. After assessing that fluorescence values remained stable, bacteria were treated with 10 μL/well of peptide, antibiotic or water. Fluorescence was quantified 1 h after treatment under the same conditions and represented as a function of peptide concentration, subtracting the background of untreated cells.

To assess bacterial survival after treatment, supernatants from the fluorescence assays were serial diluted and plated for CFU enumeration. Since neither DiSC_3_(5) nor PI affected bacterial growth or treatment susceptibility under our experimental conditions, supernatants from wells with the same treatment but different dyes were pooled together. Recovered bacterial counts (CFU/mL) were represented as a function of peptide concentration.

### 4.14. Agarose Gel Electrophoretic Mobility Shift Assay

To investigate the DNA binding capacity of As-CATH8, vancomycin and polymyxin B, an agarose gel electrophoretic mobility shift assay was performed as previously described [41]. Briefly, overnight cultures of *E. coli* BL21 Star cells harboring the pET28a plasmid were grown in LB supplemented with kanamycin (30 μg/mL). The pET28a plasmid was purified using the QIAprep Spin Miniprep Kit (QIAGEN Inc., Hilden, Germany) and linearized with the *Sma*I endonuclease (Thermofisher Scientific, Waltham, MA, USA). Linear pET28a was purified from an agarose gel using the QIAquick Gel Extraction Kit (QIAGEN Inc., Hilden, Germany).

Twofold decreasing amounts of As-CATH8 or antibiotics were incubated for 1 h at room temperature with 100 ng of linear pET28a in 10 μL of binding buffer (5% glycerol, 10 mM Tris-HCl pH 8.0, 1 mM EDTA, 1 mM DTT, 20 mM KCl and 50 μg/mL BSA). After this, 2 μL of 6× DNA Loading Dye (Thermofisher Scientific, Waltham, MA, USA) was added and the mixture was loaded onto a 1% agarose gel in TAE buffer containing SYBR Safe (Thermofisher Scientific, Waltham, MA, USA). The gel was run at 100 V for 1 h and the GeneRuler 1 kb DNA ladder (Thermofisher Scientific, Waltham, MA, USA) was used as a molecular weight marker. Finally, the gel was imaged on a ChemiDoc Touch Imaging System (BioRad Laboratories, Montreal, QC, Canada).

### 4.15. Statistical Analysis

Statistical processing was performed in RStudio using the R packages DescTools version 0.99.44 and rstatix version 0.7.0 [90]. Data normality was assessed using visual methods (Q-Q and density plots), as well as with the Shapiro–Wilk statistical test. Homogeneity of variance was analyzed using residual plots and Levene’s statistical test. The parametric ANOVA test was used for comparison between groups, followed by Tukey’s post hoc test. The Kruskal–Wallis test was used as a non-parametric method, followed by Dunn’s multiple comparison test with the Benjamini–Hochberg *p*-value correction. In all cases, *p*-values < 0.05 were considered statistically significant.

## Figures and Tables

**Table 1:** Physicochemical properties of synthetic croCATHs and human LL-37. Properties were calculated using the Peptides and modlAMP packages in R and Python, respectively.

Name	Sequence	Length	MW	Charge	HI	HM
As-CATH7	KRVNWRKVGRNTALGASYVLSFLG	24	2693	4.76	−0.15	0.25
As-CATH8	KRVNWAKVGRTALKLLPYIFG	21	2431	4.76	0.06	0.29
Gg-CATH5	TRRKWWKKVLNGAIKIAPYILD	22	2670	4.76	−0.39	0.40
Gg-CATH7	KRVNWRKVGLGASYVMSWLG	20	2308	3.76	−0.11	0.23
LL-37	LLGDFFRKSKEKIGKEFKRIVQRIKDFLRNLVPRTES	37	4493	5.76	−0.72	0.56

As: A. sinensis, Gg: G. gangeticus. Length: number of amino acid residues; MW: theoretical molecular weight in daltons, rounded values; charge: net charge according to the Bjellqvist; HI: hydrophobicity index according to the Kyte–Doolittle scale scale; HM: mean hydrophobic moment (amphipathicity), numerical values of the vectors shown in the wheel representations in Figure 2.

**Table 2:** Antimicrobial activity of the crocCATHs. The MIC values of the synthetic peptides were determined in a microdilution assay in MHB. The antibiotic vancomycin was used against Gram-positive *S. aureus* and *E. faecium* whereas polymyxin B was employed against the remaining Gram-negative strains. Values in μg/mL can be found in Supplementary Table S4.

Bacteria	MIC in μM					
	As-CATH7	As-CATH8	Gg-CATH5	Gg-CATH7	Polymyxin B	Vancomycin
*E. cloacae*	0.5	0.5	0.5	2	0.5	n.d.
*S. aureus*	4	0.5	1	16	n.d.	0.5
*K. pneumoniae*	1	0.5	0.5	4	0.5	n.d.
*A. baumannii*	0.25	0.25	0.5	1	0.5	n.d.
*P. aeruginosa*	4	1	1	8	0.5	n.d.
*E. faecium*	>64	>64	>64	64	n.d.	>64
*E. coli*	2	1	4	4	0.5	n.d.
*S. Typhimurium*	2	1	0.5	4	1	n.d.
*P. vulgaris*	>64	4	8	>64	>64	n.d.

n.d.: not determined.

**Table 3:** Biofilm inhibitory activity of the crocCATHs. The MBIC_95_ values of the synthetic peptides were determined by a microdilution assay using crystal violet to stain the adhered bacterial biomass. MBIC_95_ was defined as the minimal peptide concentration capable of inhibiting mean biofilm growth by at least 95% compared to the untreated control. Values in μg/mL can be found in Supplementary Table S5.

Bacteria	MBIC_95_ in μM			
	As-CATH7	As-CATH8	Gg-CATH5	Gg-CATH7
*E. cloacae*	32	4	4	16
*S. aureus*	4	1	1	4
*A. baumannii*	1	0.5	0.5	1
*P. aeruginosa*	>64	64	32	>64
*E. coli*	32	1	32	32
*S. Typhimurium*	1	1	1	4

## Data Availability

Sequences generated as part of this study with their NCBI accession numbers can be found in Supplementary Table S1. Accession numbers of the vertebrate sequences included in the phylogenetic analysis are included in Supplementary Table S3.

## Supplementary Materials

The following supporting information can be downloaded at: https://www.mdpi.com/article/10.3390/antibiotics11111603/s1. Table S1: Cathelicidin sequences identified in *Alligator mississippiensis*, *Alligator sinensis*, *Crocodylus porosus*, *and Gavialis gangeticus*; Table S2: Summary of prediction of identified cathelicidin sequences from *A. mississippiensis*, *A. sinensis*, *C. porosus*, and *G. gangeticus*; Table S3: Vertebrate cathelicidins peptides included in the phylogenetic analysis; Table S4: Antimicrobial activity of the crocodylian cathelicidins; Table S5: Biofilm inhibitory activity of the crocodylian cathelicidins; Figure S1: Multiple sequence alignment of novel and previously identified crocodylian cathelicidins; Figure S2: Biofilm inhibitory activity of crocodylian cathelicidin peptides against different bacterial pathogens; Figure S3: Cytotoxic activity of crocodylian cathelicidins against human cells; Figure S4: Bacterial membrane depolarization and permeabilization capacity of As-CATH8; Figure S5: DNA binding activity of As-CATH8 [18,19,91].
