# Supplementary material for: Novel Alligator Cathelicidin As-CATH8 Demonstrates Anti-Infective Activity against Clinically Relevant and Crocodylian Bacterial Pathogens
Source: Antibiotics (Basel). 2022 Nov 11;11(11):1603. doi: 10.3390/antibiotics11111603 (PMC9686568; doi:10.3390/antibiotics11111603)
Supplement: Supplementary file 1 [file antibiotics-11-01603-s001.zip › antibiotics-1969606-supplementary.pdf]

Supplementary Information

Novel alligator cathelicidin As-CATH8 demonstrates anti-infective activity against clinically relevant and crocodylian bacterial pathogens

Felix L. Santana[1,2], Karel Estrada[3], Morgan A. Alford[2], Bing C. Wu[2], Melanie Dostert[2], Lucas Pedraz[2], Noushin Akhoundsadegh[2], Pavneet Kalsi[2], Evan F. Haney[2], Suzana K. Straus[4], Gerardo Corzo[1,*], and Robert E. W. Hancock[2,*]

[1] Departamento de Medicina Molecular y Bioprocesos, Instituto de Biotecnología, Universidad Nacional Autónoma de México, Cuernavaca, 62210, Morelos, Mexico.

[2] Centre for Microbial Diseases and Immunity Research, University of British Columbia, Vancouver, V6T 1Z4, BC, Canada.

[3] Unidad de Secuenciación Masiva y Bioinformática, Instituto de Biotecnología, Universidad Nacional Autónoma de México, Cuernavaca, 62210, Morelos, Mexico.

[4] Department of Chemistry, University of British Columbia, Vancouver, V6T 1Z1, BC, Canada.

[*] Correspondence: gerardo.corzo@ibt.unam.mx (GC); bob@hancocklab.com (REWH)

Keywords: cathelicidins; antimicrobial peptides; LL-37; biofilms; abscess model; skin model; reptiles

Supplementary Tables

Table S1 Cathelicidin sequences identified in *Alligator mississippiensis*, *Alligator sinensis*, *Crocodylus porosus*, and *Gavialis gangeticus* 3

Table S2 Summary of prediction of identified cathelicidin sequences from *A. mississippiensis*, *A. sinensis*, *C. porosus*, and *G. gangeticus* 6

Table S3 Vertebrate cathelicidins peptides included in the phylogenetic analysis . . 7

Table S4 Antimicrobial activity of the crocodylian cathelicidins 8

Table S5 Biofilm inhibitory activity of the crocodylian cathelicidins 9

Supplementary Figures

Figure S1 Multiple sequence alignment of novel and previously identified crocodylian cathelicidins 10

Figure S2 Biofilm inhibitory activity of crocodylian cathelicidin peptides against different bacterial pathogens 11

Figure S3 Cytotoxic activity of crocodylian cathelicidins against human cells 12

Figure S4 Bacterial membrane depolarization and permeabilization capacity of As-CATH8 13

Figure S5 DNA binding activity of As-CATH8 14

Supplementary Tables

Table S1: Cathelicidin sequences identified in *Alligator mississippiensis*, *Alligator sinensis*, *Crocodylus porosus* and *Gavialis gangeticus*. Previously described alligator cathelicidins AM-36 [1] (renamed here as Am-CATH4) and As-CATH1-6 [2] were also recovered in our analysis and are included as a reference. Putative elastase cleavage site that indicates the start of the mature sequence is denoted with an arrow (↓) and was inferred based on protein sequence alignment with As-CATH1-6 [2]. NCBI accession numbers and references are shown.

Name	Sequence	Accession
Am-CATH1	MGRWGWGVLLGLAMLVASASASQHKMLSYEEAVSLAVDFYNQEPGIDHAFRLLRA DPQPAWDMTSKPRQELQFVVRETVCPRAQDPPASECDFKDNGLVRNCTGLFSMER ESPTVIITCDTVTPEQHVRV↓TRSERRNGHKRRRGSGSRRGQYSSTKHGGRKHP RKRPGSGSRLGHETPHVAPIDKGHVG	OL889930
Am-CATH3	MGCRGWGVLLGLATLVAAASASQRKMLTYREAALFAVDFYNRQPGIDHTFQLLSV EPQPVPDTTSKSRQEVRFVVCETVCPQADNHPDSECNIKFNGLVRNCMWLLSTEH KLPTSIITCDTVTPGKHV↓PANPKLKPGKGPGRHPSSGIWRGQGTPFSLPITKK PVG	OL889932
Am-CATH4	PDRV↓RRGLFKKLRRKIKKGFKKIFKRLPPIGVGVSIPLAGKR	Barksdale et al., OL889933
Am-CATH5	METCLCLLLLLGVATAVATAQAQAQAQTQSQSQAGYEDAVTTAVDIFNQESGLPQ AYRLLEAEPQSEWNPSSQAAQPLKFSVKETVCPIAQKGNLKQCDFKENGLVKDCS GLFTAGKKPPVTAVKCEDTSQEPELV↓TRRKFWKKILNGALKIAPFFLG	OL889934
Am-CATH6	MKSCWALVLLVGCMASAATAQSQLNFNEAVSLAVDFYNRGLAVNNTFQLLRTAPS GDVVSSPSEFRRLNFTIMETTCPVGSQPTQELCQFKENGLVRACVGFFSTQQVAP LIVVTCEEAPSEPV↓RVTRWLWLLRGGLKAAGWGIRAHLNRNQ	OL889935
Am-CATH8	APPAPALSSYQEALAAAVNTYNQESGLPQAYRLLEAEPQPQWDPSSQPAQPLKFS IKETECLVSEKRDVSQCPFKDKGLVKDCKGLYAEEKEPPVITAKCEDAGQEPQLV ↓KRVNWPKVGRTVLRFLPYILGG	OL889936
Am-CATH11	MLVASASASQHKMLSYEEAVSLAVDFYNQEPGIDHAFRLLRADPQPAWDMTSKPR QELQFVVRETVCPRAQDPPASECDFKDNGLVRNCTGLFSMERESPTVIITCDTVT PEQHVRV↓RRSGWWNRSKRRRGSGSRRGRFSHIAHGGRKGHERIARV	OL889931

As-CATH1	MGRWGWGVLLGLAMLVASASASQHKTLSYEEAVSLAVDFYNQEPGIDHAFRLLRADPQPAWDMTSKPRQELRFVVRETVCPRAQDPPASECDFKDNGLVRNCTGLFSTERESPTVIITCDTVTPGQHVRV↓RRSGWWNGHKRRRGSGSRHGQYSSTKYGGRKRPRKRPGSGSWLSHDTPHVAPIAKGHVG	Chen et al., ASN73761
As-CATH2	MGRWGWGVLLGLATFVAVALGSQHKTLSYEEAVSLAVDFYNQGPGIDHAFRLLRADPQPAWDMTSKPRQELRFVVRETVCPRAQDPPASECDFKDNGLVRNCTGLFSTERESPTVIITCDTVTPGQHVRV↓RRSGWWNGHKRRRGSGTRRGRFSHIAHGGRKGHERIA	Chen et al., ASN73762
As-CATH3	MGRWGWRVLLVLATLVAAASASQRKMLTYGEAASFAVDFYNQQPGIDHTFQLLCVEPQPVWDTTSKSRQEVRFVICETVCPQASNHPASECNLKFNGLVRNCTWLFSTEQKSPTSIITCNTVSPGKHV↓PAKPKPRPGKLSSFTLHLAPGSDGKPRCHYP	Chen et al., ASN73763
As-CATH4	MQTCWVILLLPLLGAASTELPTPGTDPPQLTPTYAQALATAVDVYNQGPGVDFAFRLLEAESRDDWDASTDPLRQLEFTLKETECPVGEDQPLDQCDFKDGGAVLDCTGTFSCSEASLMVLVTCQPAEPLPDRV↓RRGLFKKLRRKIKKGFKKIFKRLPPVGVGVSIPLAGRR	Chen et al., ASN73764
As-CATH5	METCLRLLLLLGVATAVATAQAQAQAQTQSQSQAGYEDAVTTAVDIFNQESGLPQAYRLLEAEPQSEWNPSSQAAQPLKFSVKETVCPIAQKGNLKQCDFKENGLVKDCSGLFTAGKKPPVTAVKCEDTSQEPELV↓TRRKFWKKVLNGALKIAPFLLG	Chen et al., ASN73765
As-CATH6	MKSCWALVLLVGCMASAATAQSQLNFNEAVSLAVDFYNRGLAVNNTFQLLRTAPSGDVVSSPFEFRRLNFTIMETTCPVGGQPTQGPCQFKENGLVRACVGFFSAQQVAPLIVVTCEEAPSEPVRV↓TRWLWLLRGGLKAAGWGIRAHLNRNQ	Chen et al., ASN73766
As-CATH7	NPPQDLASQPVQPLKFSIKETECLVSEKRDISQCPFKDKGLVKDCAGIYSAEKKPPIVTANCEDAGQGPQLV↓KRVNWRKVGRNTALGASYVLSFLG	OL889937
As-CATH8	NPPQDLASQPVQPLKFSIKETECLVSEKRDISQCPFKDKGLVKDCKGLYAEEKEPPVITAKCEDAGQEPQLV↓KRVNWAKVGRTALKLLPYIFG	OL889938
Cp-CATH3	MGRRGWVVLLGLATLVAAALASQRKLLSYGEAASFAVDFYNQQPGVDHTFRLLDVEPQPAWDTMAKSCQEVRFVVRETVCPWTHKLPASECDFKDNGLVRNCTWLFSTKLKLPASIITCDTMTPGKDI↓HAKPKPKPGKDERGRPGSGSWIGKGTPFSFPITKKPVG	OL889940
Cp-CATH5	MGTCLRLLLVLGVATAGLTPPARAQAQAQAQAQAQASYETAVATAVDIYNQEPGLAQAYRLLEAEPQDSWNPASQAAQPLKFTVKETTCPIAQKGNLQQCDFKENGLIKDCSGLFTAGKKPPVTDVKCVDASQEPELV↓TRKKWWKKVLKGAIKTALK	OL889941

Cp-CATH7	AFPRTPQDLASQPVQPLRFSIKETTCLVSEKRDVRQCPFKDEGLVKDCTGIYSAE KKPPIVTAVCKDAGQEPELV↓KRVNWRKIGLGASYVMSWLG	OL889942
Cp-CATH10	MGRRGWVVLLALATLAAGALGSQHRTLSYEEAVSLAVNFYNQGPGIDHAFRLLRA DPQPAWDMTSQPRQELRFVVRETVCPRAQDPPASECDFQDNGLVRNCTGLFSTER ESPTVIITCDTATPGQHARV↓RRSGWRDRIKRHRGSDSRLGYMTPFSTNINKG	OL889939
Gg-CATH2	MGRRGWVVLLGLATLVAVALGSQHKTLSYEEAVSLAVSFYNQGPGIDRAFRLLRA DPQPAWDMTSKPRQELRFVVRETVCPRAQDPPASECDFKDHGLVRNCTGLFSMER ESPTVIITCDTVTPGQHVRV↓RRSGWWDWIKGRRGSGSRRGRFTHIAHGGSKGH GNNGNIA	OL889943
Gg-CATH4	MQTCWVVLLLLLPLLGAASTDLPTPGTDLPQPMPSYAQALATAVDVYNQGSGVDA AFQVLEAESRDDWDANEDPLRQLGFRLKETECPVGEDQPLDQCDFKDGGAVLDCT ATFSCSEASLMVLVTCQPAEPPPTRD↓RRGLFKKLRRKIKKAFKKVFKRLPPVG VGVSIPLSGHR	OL889944
Gg-CATH5	SPQNPSSQAAQPLKFSVKETVCPIAQKGNLQQCNFKENGLIKDCSGLFTAAKKPP VTDVKCVDASQEPELV↓TRRKWWKKVLNGAIKIAPYILD	OL889945
Gg-CATH6	MKSCWALVVLVGCMASTAAAQSQLNFNEAVSLAVDFYNRGLAVNNTFQLLRTAPE GDVVSKPFEFRRLNFTIMETTCPVAGQLPSEPCQFKENGLVRACVGFFSAQQVAP LIVVTCEEAPSEPVRV↓TRWIGLVRGGLKLAGWGLRTYLNRNR	OL889946
Gg-CATH7	MQPCLRALLVLGAAVAVAMAVALPAPPAPAPKGYQEALAAAINTYNQESGLPQAY RLLEAEPQPQWDPASQPVQPLKFSIKETTCLVSEKRDVSQCLFKDKGLVKDCTGI YSVEKQPPIVTAVCKDAGQEPQLV↓KRVNWRKVGLGASYVMSWLG	OL889947
Gg-CATH9	MGRRGWVVLLGLATLVAVALGSQHKTLSYEEAVSLAVSFYNQGPGIDRAFRLLRA DPQPAWDMTSKPRQELRFVVRETVCPRAQDPPASECDFKDHGLVRNCTGLFSMER ESPTVIITCDTVTPGQHVRV↓RRSGRWDWIKGRRGSGSRRGHYSTIRRGGRKGS GR	OL889948

Am: *A. mississippiensis*; As: *A. sinensis*; Cp: *C. porosus*; Gg: *G. gangeticus*

Table S2: Summary of prediction of identified cathelicidin sequences from *A. mississippiensis*, *A. sinensis*, *C. porosus*, and *G. gangeticus*. Previously described alligator cathelicidin sequences Am-CATH4 [1] and As-CATH1-6 [2] were also recovered in the analysis and are included in this table as a reference.

Name	Genome assembly	Strand	Start	Stop	Missing region	References
Am-CATH1	gi_397460297_gb_JH735204	-	83125	93242	No	-
Am-CATH3	gi_397456510_gb_JH738990	-	0	6010	No	-
Am-CATH4	gi_397462851_gb_JH732650	-	60	12217	N-ter	Barksdale et al.
Am-CATH5	gi_397462851_gb_JH732650	-	24092	34308	No	-
Am-CATH6	AKHW03001069	-	5231	15336	No	-
Am-CATH8	AKHW03001069	-	33404	42943	N-ter	-
Am-CATH11	AKHW03000909	+	2068	12185	N-ter	-
As-CATH1	gi_555310491_ref_NW_005843086	-	367921	378020	No	Chen et al.
As-CATH2	gi_555311385_ref_NW_005842192	+	460	10559	No	Chen et al.
As-CATH3	gi_555311385_ref_NW_005842192	+	5263	15383	No	Chen et al.
As-CATH4	gi_555310491_ref_NW_005843086	-	0	6074	No	Chen et al.
As-CATH5	gi_555311385_ref_NW_005842192	+	25222	35432	No	Chen et al.
As-CATH6	gi_555311385_ref_NW_005842192	+	53173	63350	No	Chen et al.
As-CATH7	gi_555311385_ref_NW_005842192	+	12151	22328	N-ter	-
As-CATH8	gi_555311385_ref_NW_005842192	+	16243	26462	N-ter	-
Cp-CATH3	gi_698053534_gb_JRXG01102469	-	0	6985	No	-
Cp-CATH5	gi_698089567_gb_JRXG01066436	+	4753	14996	No	-
Cp-CATH7	gi_698089567_gb_JRXG01066436	+	0	5837	N-ter	-
Cp-CATH10	gi_698084222_gb_JRXG01071781	-	2052	12151	C-ter	-
Gg-CATH2	gi_699340657_gb_JRWT01256782	-	0	2350	No	-
Gg-CATH4	gi_699490068_gb_JRWT01141868	+	6112	13962	No	-
Gg-CATH5	gi_699490071_gb_JRWT01141865	+	0	4273	N-ter	-
Gg-CATH6	gi_699490068_gb_JRWT01141868	+	0	7535	No	-
Gg-CATH7	gi_699518732_gb_JRWT01120362	+	0	7133	No	-
Gg-CATH9	gi_699359619_gb_JRWT01242099	+	7	10103	C-ter	-

-: in references denotes the current study

Table S3: Vertebrate cathelicidins peptides included in the phylogenetic analysis. Scientific and common names for each species, as well as the cathelicidin peptide names and NCBI accession numbers are listed. Previously described alligator cathelicidins and all crocodylian cathelicidins identified in this study are shown in Table S1.

Vertebrate group	Species	Common name	Peptide	Accesion
Amphibian	*Odorrana livida*	Green cascade frog	Ol-CATH2	AXR75914
	Bufo gargarizans	Asiatic toad	Bg-CATH	ANV28414
Bird	*Gallus gallus*	Chicken	Gga-CATH1	NP_001001605
	Phasianus colchicus	Ring-necked pheasant	Pc-CATH3	ACZ45041
	Coturnix japonica	Japanese quail	Cj-CATH2	BAW94547
	Coturnix coturnix	Common quail	Cj-CATH3	ACZ45043
	Columba livia	Rock pigeon	Cl-CATH	XP_005512199
	Anas platyrhynchos	Mallard	Ap-CATH	ALD83753
	Gallus gallus	Chicken	Gga-CATH2	NP_001020001
	Cygnus atratus	Black swan	Ca-CATH	XP_035410280
	Gallus gallus	Chicken	Gga-CATH3	NP_001298106
Reptile	*Naja atra*	Chinese cobra	Na-CRAMP	B6S2X0
	Bothrops atrox	Barba amarilla	Batroxicidin	U5KJC9
	Hydrophis cyanocinctus	Asian annulated sea snake	Hc-CATH	AKJ54480
	Python bivittatus	Burmese python	Pb-CATH	XP_007443270
	Python bivittatus	Burmese python	Pb-CATH	XP_007442672
	Pelodiscus sinensis	Chinese soft shelled turtle	Ps-CATH2	XP_006114479
	Trimerodytes annularis	Red-bellied annulate keelback	Ta-CATH	QBZ68899
	Chelonia mydas	Green sea turtle	Cm-CATH2	XP_007066511
	Chelonia mydas	Green sea turtle	Cm-CATH	XP_007066509
	Pseudonaja textilis	Tropical rattlesnake	Pt-CATH1	U5KJJ1
	Bungarus fasciatus	Banded crait	Bf-CATH	B6D434
	Python bivittatus	Python	Pb-CATH	XP_007445262
	Pelodiscus sinensis	Chinese soft shelled turtle	Ps-CATH3	AWW22372
	Crocodylus siamensis	Siamese crocodile	Cs-CATH	AYV89837
	Mus musculus	House mouse	Mm-CATH	AAB88303
	Oryctolagus cuniculus	Rabbit	Oc-CATH	NP_001075774

	Pan paniscus	Bonobo	Pp-CATH	XP_003818474
	Canis lupus familiaris	Dog	Clu-CATH	NP_001003359
	Ovis aries	Sheep	Bactinecin-11	AAB62000
	Equus caballus	Horse	Ec-CATH3	NP_001075399
Mammal	*Sus scrofa*	Pig	Protegrin-4	NP_999028
	Trachypithecus cristatus	Silvery lutung	Tc-CATH	B6S2X2
	Sarcophilus harrisii	Tasmanian devil	Sh-CATH2	XP_012398889
	Homo sapiens	Human	LL-37	NP_004336
	Bos taurus	Cattle	BMAP-27	NP_777257
	Macaca mulatta	Rhesus monkey	Mmul-CATH	NP_001028681
	Trichechus manatus latirostris	Florida manatee	Tm-CATH2	XP_023586559

Table S4: Antimicrobial activity of the crocodylian cathelicidins. The MIC values of the synthetic peptides were determined in a microdilution assay in MHB. The antibiotic vancomycin was used against Gram-positive *S. aureus* and *E. faecium* whereas polymyxin B was employed against the remaining Gram-negative strains.

	MIC in μM (μg/mL)						
**Bacteria**	**As-CATH7**	**As-CATH8**	**Gg-CATH5**	**Gg-CATH7**	**LL-37**	**Polymyxin B**	**Vancomycin**
E. cloacae	0.5 (1.3)	0.5 (1.2)	0.5 (1.3)	2 (4.6)	n.d.	0.5 (0.6)	n.d.
S. aureus	4 (10.8)	0.5 (1.2)	1 (2.7)	16 (37)	n.d.	n.d.	0.5 (0.7)
K. pneumoniae	1 (2.7)	0.5 (1.2)	0.5 (1.3)	4 (9.2)	n.d.	0.5 (0.6)	n.d.
A. baumannii	0.25 (0.7)	0.25 (0.6)	0.5 (1.3)	1 (2.3)	n.d.	0.5 (0.6)	n.d.
P. aeruginosa	4 (10.8)	1 (2.4)	1 (2.7)	8 (18.5)	n.d.	0.5 (0.6)	n.d.
E. faecium	>64 (>172)	>64 (>156)	>64 (>171)	64 (148)	n.d.	n.d.	>64 (>93)
E. coli	2 (5.4)	1 (2.4)	4 (10.7)	4 (9.2)	8 (36)	0.5 (0.6)	n.d.
S. Typhimurium	2 (5.4)	1 (2.4)	0.5 (1.3)	4 (9.2)	16 (72)	1 (1.2)	n.d.
P. vulgaris	>64 (>172)	4 (9.7)	8 (21)	>64 (>148)	n.d.	>64 (>77)	n.d.

n.d.: not determined

Table S5: Biofilm inhibitory activity of the crocodylian cathelicidins. The $MBIC_{95}$ values of the synthetic peptides were determined by a microdilution assay using crystal violet to stain the adhered bacterial biomass. $MBIC_{95}$ was defined as the minimal peptide concentration capable of inhibiting mean biofilm growth by at least 95% compared to the untreated control.

	**$MBIC_{95}$ in μM (μg/mL)**			
**Bacteria**	**As-CATH7**	**As-CATH8**	**Gg-CATH5**	**Gg-CATH7**
E. cloacae	32 (86)	4 (9.7)	4 (10.7)	16 (37)
S. aureus	4 (10.8)	1 (2.4)	1 (2.7)	4 (9.2)
A. baumannii	1 (2.7)	0.5 (1.2)	0.5 (1.3)	1 (2.3)
P. aeruginosa	>64 (>172)	64 (156)	32 (85)	>64 (>148)
E. coli	32 (86)	1 (2.4)	32 (85)	32 (74)
S. Typhimurium	1 (2.7)	1 (2.4)	1 (2.7)	4 (9.2)

Supplementary Figures

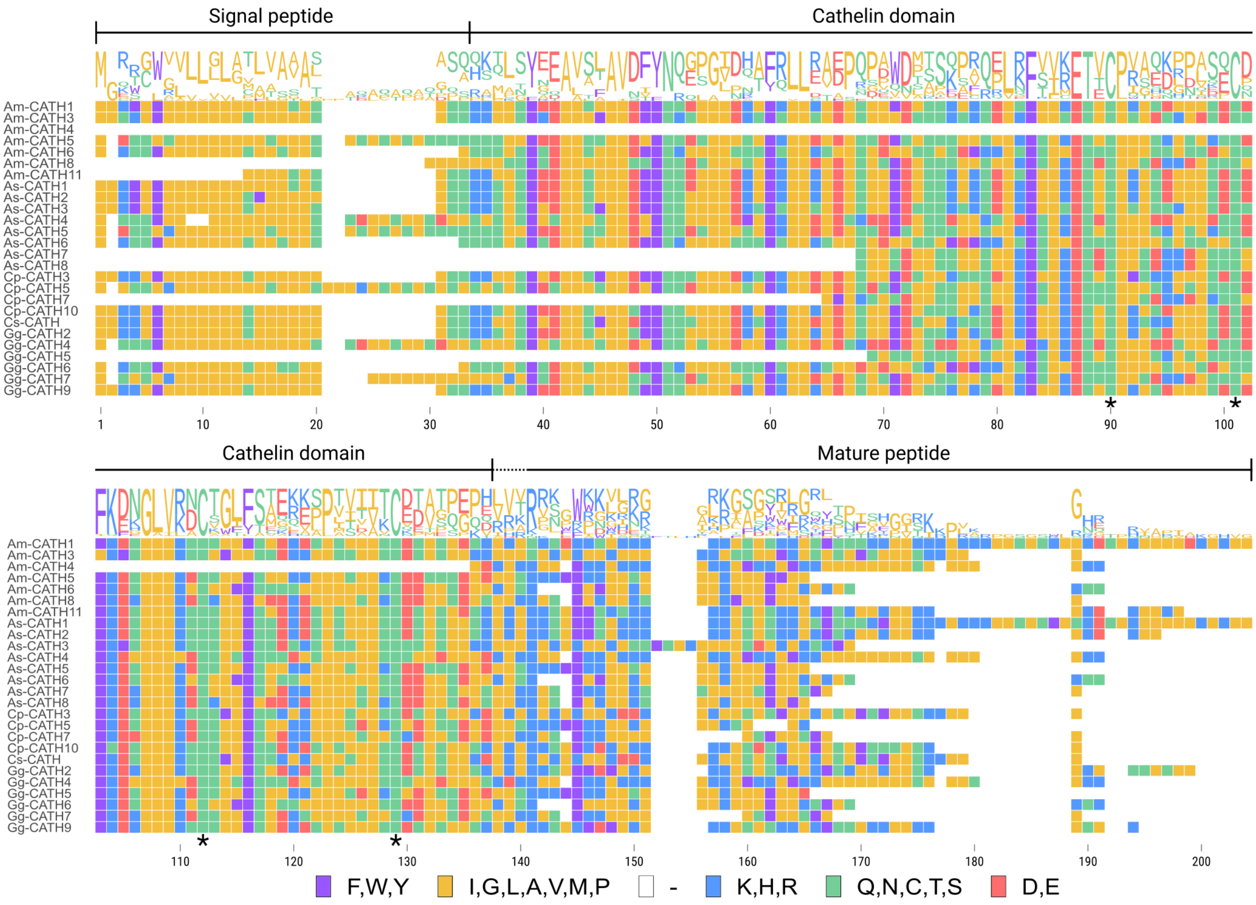

Figure S1: Multiple sequence alignment of novel and previously identified crocodylian cathelicidins. Crocodylian cathelicidins showed the three classical domains present in the cathelicidin family of host defense peptides (HDPs): the signal peptide, cathelin domain, and mature peptide region. The sequences were aligned using the MAFFT algorithm and visualized in R with the package ggmsa version 1.0.3. Amino acid residues are colored according to the Chemistry-AA coloring scheme. Highly conserved cysteines in the cathelin domain are marked with black asterisks (*). Previously described cathelicidins Am-CATH4 [1], As-CATH1-6 [2], and Cs-CATH [3] were also included. Putative elastase cleavage site to process the mature peptide is located within residues 138-140 depending on the sequence. Abbreviations: Am: *A. mississippiensis*; As: *A. sinensis*; Cp: *C. porosus*; Cs: *C. siamensis*; Gg: *G. gangeticus*.

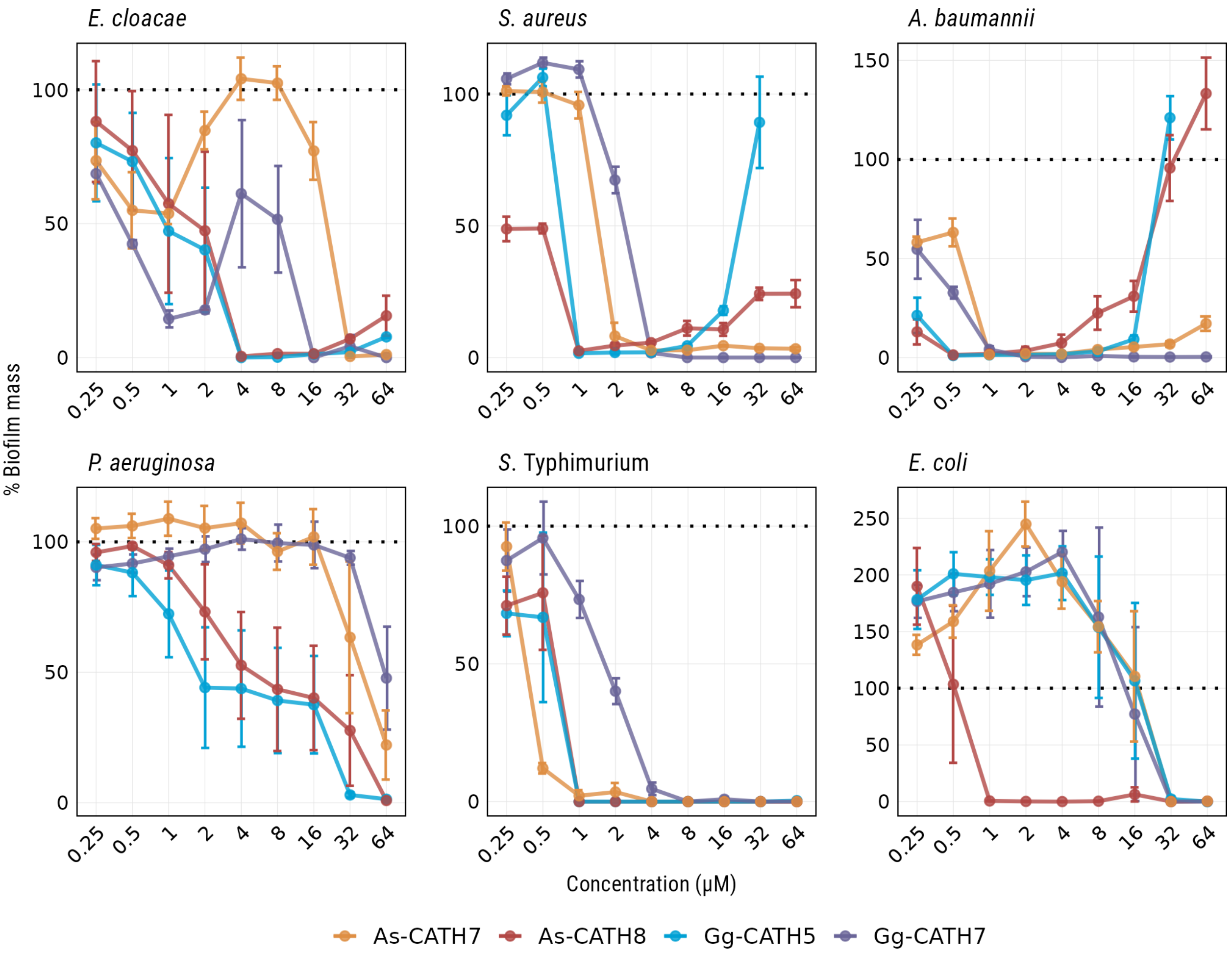

Figure S2: Biofilm inhibitory activity of crocodylian cathelicidin peptides against different bacterial pathogens. Dose-response curves were obtained in different media depending on the bacterial strain and, after 24 h treatment, the adhered biofilm mass was stained with crystal violet. Results are displayed as percentage of biofilm mass with respect to the growth control (defined as 100%). The mean $\pm$ standard error of the mean of at least three biological replicates is shown. Cathelicidins As-CATH8 and Gg-CATH5 enhanced crystal violet staining of *S. aureus* and *A. baumannii* (10% TSB supplemented with 0.1% glucose) at high concentrations. However, this is likely the result of peptide and/or dead bacteria precipitation and not due to an increase in biofilm mass since no bacterial colonies were recovered from these wells. For visualization purposes, the data point corresponding to Gg-CATH5 (64 μM) is not shown for these two bacteria.

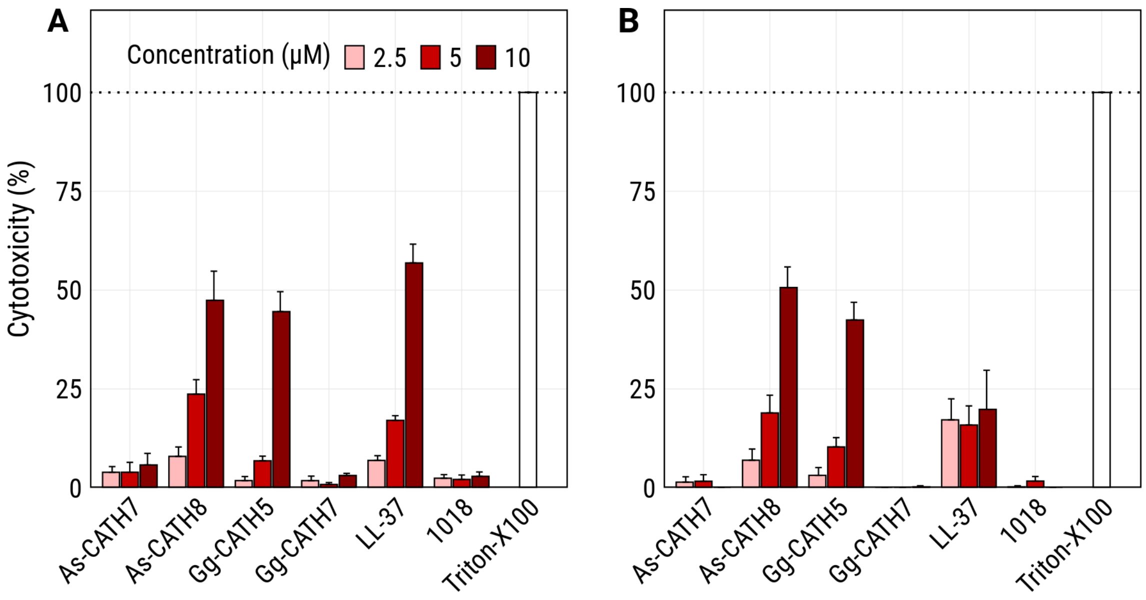

Figure S3: Cytotoxic activity of crocodylian cathelicidins against human cells. The peptide-induced cytotoxicity was measured with the LDH release assay. Values are presented as percentage of cytotoxicity relative to the positive control (cells treated with 2% Triton X-100). Mean $\pm$ standard error of six biological replicates is shown. Human cathelicidin LL-37 and immunomodulatory peptide 1018 were included as a comparison and control, respectively. **A:** Cytotoxicity against the human bronchial epithelial (HBE) cell line. **B:** Cytotoxicity against human peripheral blood mononuclear cells (PBMCs).

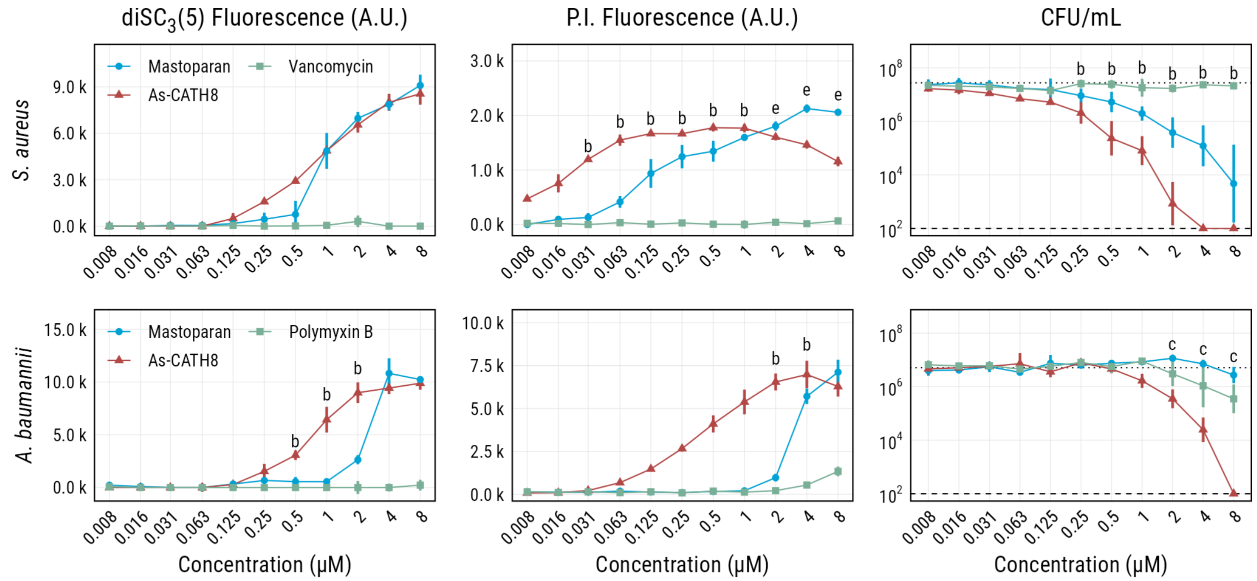

Figure S4: Bacterial membrane depolarization and permeabilization capacity of As-CATH8. The activity against *S. aureus* and *A. baumannii* planktonic cells were evaluated using $diSC_3(5)$ and propidium iodide (PI) dyes, revealing alterations of membrane potential and permeabilization of cells, respectively. The membrane-permeabilizing peptide Mastoparan was used as positive control. Fluorescence readings (arbitrary units), after 1 h treatment against planktonic cells, are shown as mean $\pm$ standard error, whereas CFU data is presented as geometric mean $\times/$ geometric standard deviation. The geometric mean of the CFU counts corresponding to the untreated control is shown as dotted lines. Peptidic antibiotics vancomycin and polymyxin B were used as a comparison for *S. aureus* and *A. baumannii*, respectively. Letters denote statistically significant differences ($p < 0.05$) between As-CATH8 and antibiotics (b), As-CATH8 and Mastoparan (c) or antibiotics and Mastoparan (e), according to the Kruskal-Wallis test followed by the Dunn's post hoc test with the Benjamini-Hochberg correction for multiple comparisons. Experiments were performed at least three times.

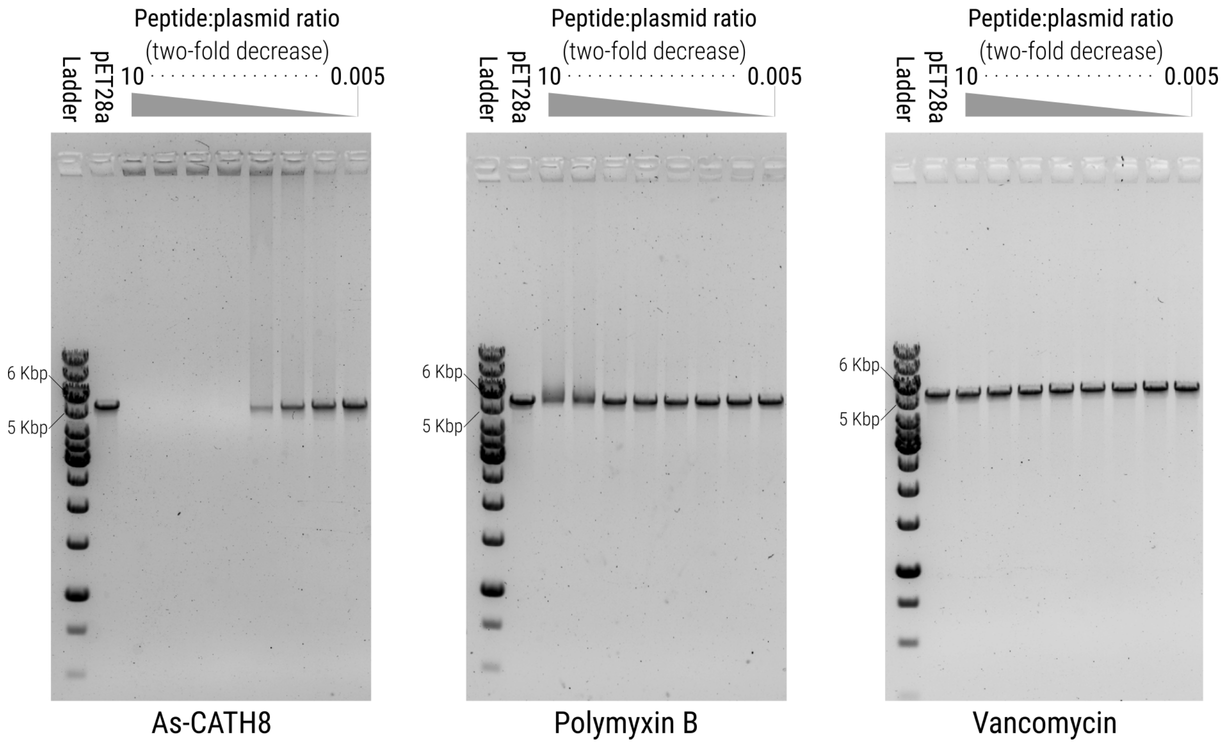

Figure S5: DNA binding activity of As-CATH8. DNA binding capacity of As-CATH8 was investigated in a gel electrophoretic mobility shift assay. As-CATH8 was incubated for 1 h at room temperature in binding buffer with 100 ng of the linearized pET28a plasmid at two-fold decreasing peptide:plasmid weight ratios. Mixtures or pure pET28a, included as a negative control, were applied to a 1% agarose gel, electrophoretically separated in TAE buffer 100 v for 1 h and stained with SYBR Safe. The GeneRuler 1 kb DNA Ladder was used as a molecular weight marker. In all the assays, the peptide antibiotics vancomycin and polymyxin B were used as comparison. Overall, the minimal ratio at which As-CATH8 impeded pET28a electrophorectic migration was 1.25, whereas partial effects were observed at 0.625. Experiments were performed at least three times and a representative image is shown.

Supplementary References

1. Barksdale, S.M.; Hrifko, E.J.; van Hoek, M.L. Cathelicidin Antimicrobial Peptide from *Alligator Mississippiensis* Has Antibacterial Activity Against Multi-Drug Resistant *Acinetobacter Baumanii* and *Klebsiella Pneumoniae*. *Dev. Comp. Immunol.* **2017**, *70*, 135–144, doi:10.1016/j.dci.2017.01.011.
2. Chen, Y.; Cai, S.; Qiao, X.; Wu, M.; Guo, Z.; Wang, R.; Kuang, Y.-Q.; Yu, H.; Wang, Y. As-Cath1-6, Novel Cathelicidins with Potent Antimicrobial and Immunomodulatory Properties from *Alligator Sinensis*, Play Pivotal Roles in Host Antimicrobial Immune Responses. *Biochem. J.* **2017**, *474*, 2861–2885, doi:10.1042/BCJ20170334.
3. Tankrathok, A.; Punpad, A.; Kongchaiyapoom, M.; Sosiangdi, S.; Jangpromma, N.; Daduang, S.; Klaynongsruang, S. Identification of the First *Crocodylus Siamensis* Cathelicidin Gene and Rn15 Peptide Derived from Cathelin Domain Exhibiting Antibacterial Activity. *Biotechnol. Appl. Biochem.* **2019**, *66*, 142–152, doi:10.1002/bab.1709.
